# TRIM8 restores p53 tumour suppressor function by blunting N-MYC activity in chemo-resistant tumours

**DOI:** 10.1186/s12943-017-0634-7

**Published:** 2017-03-21

**Authors:** Francesca Mastropasqua, Flaviana Marzano, Alessio Valletti, Italia Aiello, Giuseppe Di Tullio, Annalisa Morgano, Sabino Liuni, Elena Ranieri, Luisa Guerrini, Giuseppe Gasparre, Elisabetta Sbisà, Graziano Pesole, Antonio Moschetta, Mariano Francesco Caratozzolo, Apollonia Tullo

**Affiliations:** 1 Institute of Biomedical Technologies ITB, CNR, Bari, Italy; 2 Department of Biosciences, Biotechnologies and Biopharmaceutics, University of Bari “A. Moro”, Bari, Italy; 3 Mario Negri Sud Foundation, Santa Maria Imbaro (Chieti), Italy; 4 Department of Medical and Surgical Sciences, University of Foggia, Foggia, Italy; 5 Department of Biosciences, University of Milano, Milano, Italy; 6 Department of Medical and Surgical Sciences, University of Bologna, Bologna, Italy; 7 Institute of Biomembranes and Bioenergetics IBBE, CNR, Bari, Italy; 8 Department of Interdisciplinary Medicine, University of Bari “Aldo Moro”, Bari, Italy

**Keywords:** TRIM8, Drug resistance, N-MYC, p53, miR-17 family

## Abstract

**Background:**

TRIM8 plays a key role in controlling the p53 molecular switch that sustains the transcriptional activation of cell cycle arrest genes and response to chemotherapeutic drugs. The mechanisms that regulate TRIM8, especially in cancers like clear cell Renal Cell Carcinoma (ccRCC) and colorectal cancer (CRC) where it is low expressed, are still unknown. However, recent studies suggest the potential involvement of some microRNAs belonging to miR-17-92 and its paralogous clusters, which could include TRIM8 in a more complex pathway.

**Methods:**

We used RCC and CRC cell models for in-vitro experiments, and ccRCC patients and xenograft transplanted mice for in vivo assessments. To measure microRNAs levels we performed RT-qPCR, while steady-states of TRIM8, p53, p21 and N-MYC were quantified at protein level by Western Blotting as well as at transcript level by RT-qPCR. Luciferase reporter assays were performed to assess the interaction between TRIM8 and specific miRNAs, and the potential effects of this interaction on TRIM8 expression. Moreover, we treated our cell models with conventional chemotherapeutic drugs or tyrosine kinase inhibitors, and measured their response in terms of cell proliferation by MTT and colony suppression assays.

**Results:**

We showed that TRIM8 is a target of miR-17-5p and miR-106b-5p, whose expression is promoted by N-MYC, and that alterations of their levels affect cell proliferation, acting on the TRIM8 transcripts stability, as confirmed in ccRCC patients and cell lines. In addition, reducing the levels of miR-17-5p/miR-106b-5p, we increased the chemo-sensitivity of RCC/CRC-derived cells to anti-tumour drugs used in the clinic. Intriguingly, this occurs, on one hand, by recovering the p53 tumour suppressor activity in a TRIM8-dependent fashion and, on the other hand, by promoting the transcription of miR-34a that turns off the oncogenic action of N-MYC. This ultimately leads to cell proliferation reduction or block, observed also in colon cancer xenografts overexpressing TRIM8.

**Conclusions:**

In this paper we provided evidence that TRIM8 and its regulators miR-17-5p and miR-106b-5 participate to a feedback loop controlling cell proliferation through the reciprocal modulation of p53, miR-34a and N-MYC. Our experiments pointed out that this axis is pivotal in defining drug responsiveness of cancers such ccRCC and CRC.

**Electronic supplementary material:**

The online version of this article (doi:10.1186/s12943-017-0634-7) contains supplementary material, which is available to authorized users.

## Background

The efficacy of current cancer treatments is often limited by the development of therapeutic resistance whose mechanisms still remain not fully elucidated. This is the case of clear cell Renal Cell Carcinoma (ccRCC), the most common subtype of RCC accounting for about 80% of surgical cases. When localized, this cancer type may be curable with surgery, although a substantial number of patients have evidence of metastasis at the time of diagnosis and a relevant number develop systemic recurrence after primary tumour resection [1]. ccRCC is paradigmatic in the way that it is characterized by exceptionally high resistance to radiation and chemotherapy, despite p53 tumour suppressor gene mutations being particularly rare. In this tumour, specific p53 pathways are inhibited by mechanisms, often unknown, other than mutations of the protein itself, resulting in a phenotype characterized by tumourigenesis, chemo-resistance, invasion and metastasis, that could be reverted by acting on the inhibiting factors.

Recently, we showed that TRIM8 protein has a prominent role in regulating cancer cell growth in vivo in ccRCC [2]. TRIM8 is a member of the TRIM/RBCC protein family, characterized by the presence of a tripartite motif consisting of a **R**ING finger, one or two **B**-box and a **C**oiled **C**oil region. A peculiarity of the proteins belonging to this family is the variety of roles exerted by each of its members. This characteristic is due to their structure that enables them to fulfil both structural and functional tasks. Some TRIM proteins show a dual role either as oncogene or tumour suppressor, depending on the cellular context [3, 4]. Similarly, TRIM8 exerts more than one role in quite diverse pathways, as in embryonic development and differentiation, in innate immune response and in a variety of human cancers [5, 6]. In ccRCC, we demonstrated that TRIM8 is a direct p53 target gene that - through a feedback loop mechanism - appears to be a pivotal component in controlling the molecular switch that directs p53 toward transcriptional activation of cell cycle arrest genes, such as p21 and GADD45 [2]. Noteworthy, we found that TRIM8 expression level is significantly decreased in ccRCC compared to matched non-tumour tissue, and such a signature was typical of this more malignant neoplasms, whereas benign oncocytomas (ROs), for instance, didn’t show it [7].

In our previous paper, we demonstrated that TRIM8 deficit is associated to Loss of Heterozygosity (LOH) of the TRIM8 locus in ccRCC cell lines; however, cases of gene-dosage compensation in LOH conditions are known, therefore we could not exclude other possible mechanisms involved in TRIM8 expression reduction [7]. Among these, microRNA regulation of expression showed up as one of the most interesting, as miR-17-92 cluster targeting and regulation of TRIM8 mRNA has been recently reported [8, 9]. This cluster is located on chromosome 13q31.3 in the third intron of the C13orf25 gene, and contains 6 miRNAs (miR-17, miR-18, miR-19a, miR-20a, miR-19b-1, miR-92-1). The human genome contains two paralogues of the miR-17-92 cluster: 1) the miR-106b/25 cluster (miR-106b, miR-93, miR-25) is located on chromosome 7 (7q22.1) in the 13th intron of the Mini-Chromosome Maintenance gene MCM7); 2) the miR-106a/363 cluster (miR-106a, miR-18b, miR-20b, miR-19b-2, miR-92-2, miR-363) is located on chromosome X (Xq26.2). Together these three miRNA clusters contain a total of 15 miRNAs constituting four “seed” families: the miR-17, the miR-18, the miR-19 and the miR-92 family. While the miR-106a/363 cluster is rarely expressed in adult human tissues, the miR-17-92 and miR-106b/25 clusters are emerging as key actors in a wide range of biological processes including tumorigenesis [10–12]. An increasing number of papers reported that miR-106b-5p and miR-17-5p, above all the microRNAs of the miR-17-92 cluster, are overexpressed in many different chemo/radio-resistant cancers, including ccRCC, glioma and Colorectal Cancers (CRC). This latter is one of the most common cancers worldwide and, as in ccRCC, the poor prognosis is due to late diagnosis and low chemotherapy response. Therefore, drug resistance poses a great challenge in this widespread cancer too [9, 13–15].

The mechanisms through which the miRNAs-TRIM8-p53 axis – whose existence here we postulate based on the aforementioned observations - may impact chemo-resistance remain largely unknown. Moreover, we hypothesize the presence of an additional factor in this regulatory axis, that is N-MYC, a well-known oncogene deregulated in a myriad of human cancers and placed at the nexus of cell growth, proliferation, metabolism, and genome stability [16]. Indeed, the miR-17-92 cluster as well as the MCM7 gene, containing the miR-106b/25 cluster, are transcriptionally activated by N-MYC [17–20].

In this paper, using two independent cancer cell models, we unveiled a novel regulatory mechanism where inhibition of miR-17-5p and/or miR-106-5p leads to recover TRIM8-mediated p53 tumour suppressor activity and strong inhibition of N-MYC-dependent cell proliferation by p53-dependent N-MYC destabilization through miR-34a up-regulation. Finally, we demonstrated the influence of the N-MYC-miRNAs-TRIM8-p53 axis on the efficacy of cancer treatments in ccRCC and CRC.

## Methods

### Cells and treatments

The human proximal tubular epithelial cells HK-2 (wild type p53), the human renal cell carcinoma RCC-Shaw (ccRCC-derived cell line with wt-p53), the human renal carcinoma of BHD (Birt-Hogg-Dubè) origin UOK-257 cells (RCC cell line with mutated p53) and the colon cancer cell line HCT116 were cultured in Dulbecco’s modified Eagle’s medium (D-MEM) plus 10% foetal bovine serum (FBS), L-Glutamine (2 mM), penicillin (100 U/ml) and streptomycin (100 μg/ml) at 37 °C, 5% CO_2_. Nutlin-3 10 μM (Cayman), Cisplatin 7.5 μM (Sigma), Sorafenib 10 μM (SantaCruz) and Axitinib 10 μM (SantaCruz) were used for 24 h [2, 7, 21].

In particular, Sorafenib and Axitinib are normally used for treatment of ccRCC patients and are commercially available with the name Nexavar^®^ and Inlyta^®^, respectively.

### Transfections

2.5 × 10^5^ human HK-2, RCC-Shaw, UOK-257 or HCT116 cells were plated in 6-well plates (60–80% confluency) and transfected with 50 pmol of miR-17-5p-mimic, miR-106b-5p-mimic, anti-miR-17-5p, anti-miR-106b-5p or Negative Control miRNA Mimic (Ambion) using SiPORT NeoFX Transfection Agent (Ambion). 24 h later cells were treated with the specific chemotherapeutic drugs (Nutlin-3, Cisplatin, Sorafenib or Axitinib). 48 h after transfections cells were collected for MTT (3-(4,5-dimethylthiazol-2-yl)-2,5-diphenyltetrazolium bromide) assay or for RNA and protein extractions.

Four different TRIM8 short hairpin RNAs (Origene™) were used to silence TRIM8 transcript by adding them to the medium containing the transfection reagent and incubating the shRNAs-transfecting agent complex at room temperature for 20 min before adding it to the cell cultures for 48 h.

TRIM8 specific shRNAs are here reported:

1. TGATAAGACGGAGGATGTCAGCTTCATGA;
2. AACCTGAAGCTCACCAACATCGTGGAGAA;
3. TAAGATCGGCCACCTGAACTCCAAGCTCT;
4. CGCAAGATTCTCGTCTGTTCTGTGGACAA

### Cell proliferation assays by MTT reduction

2 × 10^5^ cells were plated in six-well plates. After treatments, 200 μl of MTT solution (5 mg/ml) were added to the cells for 4 h at 37 °C. The medium was then removed and the reduced blue formazan crystals were suspended in isopropanol prior to reading the absorbance at 580 nm. Relative cell proliferation rate was measured respect to the sample transfected with the Negative Control miRNA Mimic. The reported data represent the average of at least three independent experiments and are shown with their standard deviations. Two-tailed Student’s T tests were performed to assess the statistical significance of cell proliferation rate.

### Colony suppression assay

2.0 × 10^5^ HK-2, RCC-Shaw, UOK-257 or HCT116 cells were plated in six-well plates (50–60% confluency) and transfected with 50 pmol of anti-miR-17-5p, anti-miR-106b-5p or Negative Control miRNA Mimic (Ambion) using SiPORT NeoFX Transfection Agent (Ambion). 24 h later cells were treated with the specific chemotherapeutic drugs (Nutlin-3, Cisplatin, Sorafenib or Axitinib). 48 h after transfections cells were rinsed with PBS, fixed with methanol for 30 min at room temperature and stained with Giemsa.

### Luciferase reporter assay

To assess miRNA/target interaction, the TRIM8 3’UTR fragment containing miR-17-5p/miR-106b-5p binding site wild type or mutant (wt or mut), and the p21 3’UTR fragment containing miR-17-5p/miR-106b-5p binding site wild type (wt) were cloned into pMIR Luciferase reporter vector (Life Technologies) downstream of the reporter luciferase gene. The genomic fragments were amplified by PCR using specific primers (available upon request). Resulting clones were sequenced to verify proper sequence identity.

2.5 × 10^5^ human HK-2 or HCT116 cells were plated in six-well plates (60–80% confluency) and transfected with 50 pmol of miR-17-5p-mimic, miR-106b-5p-mimic, anti-miR-17-5p, anti-miR-106b-5p or Negative Control miRNA Mimic (Ambion), using SiPORT NeoFX Transfection Agent (Ambion). 12 h later cells were transfected with pMIR-TRIM8 3’UTR-wt, pMIR-TRIM8 3’UTR-mut or pMIR-p21 3’UTR and with the pRL SV40 renilla luciferase vector (transfection control). 42 h later, cells were lysed by using Passive-Lysis buffer (Promega). Both firefly and renilla luciferase activities were measured using the Dual-Luciferase Assay System (Promega) and quantified on a TD-20/20 Luminometer (Turner Designs). Firefly luciferase was normalized to renilla luciferase activity, presented as relative luciferase activity.

The data reported represent the average of at least three independent experiments and are shown with their standard deviations.

### In vivo xenograft tumour studies

Eighteen age- and sex-matched CD1 nude mice from Charles River Laboratories were used for these studies. 7 × 10^6^ HCT116 cells were harvested in PBS and injected subcutaneously on each flank of the nude mice (cells/flank). Four days later mice were randomized in three homogenized experimental groups; tumours thereby generated were treated with 1x10^9^ MOI (multiplicity of infection) of each of the three recombinant adenoviruses expressing HA-TRIM8, HA-RING-TRIM8 or LacZ in sterile PBS, two times per week for three weeks. The tumours were measured using Vernier calipers, and the volume was calculated using a standard formula (Width^2^ × Length × 0.5) [22, 23]. After 21 days from the first treatment mice were sacrificed and tumours were collected and weighted.

### RNA extraction from tissues and cell lines

Tumour and paired adjacent non-tumour renal parenchyma samples from a total of 24 patients were used for this work. Immediately after surgery, tissues were separately stored and frozen at -80 °C according to a standard procedure. From histological examination, 20 samples were classified as clear cell RCCs (10 males and 10 females; mean age: 63 ± 10.8 years) and 4 were oncocytomas (all males; mean age: 63.25 ± 4.86 years) (Additional file 1: Table S1). The pathological staging was determined according to the latest TNM classification and grading according to Fuhrman. Informed consent to take part in this study was obtained from all the patients. The Hospital’s Ethics Committee approved the study. Collected ccRCC samples were processed for total RNA extraction from 50 to 100 mg of fresh frozen tissue using the TRIzol reagent (Life Technologies™). All RNA samples were purified using the RNeasy Mini kit (Qiagen^®^) according to the manufacturer’s instructions.

Xenograft tumours were immediately frozen in liquid nitrogen after excision. Tissues were homogenated by Tissue Lyser II (20Hz; 2 min, repeated two times) in RLT buffer (Qiagen^®^). Total RNA was extracted using the miRNeasy kit (Qiagen^®^) according to the manufacturer’s instructions.

HK-2, RCC-Shaw, UOK-257 or HCT116 cells were plated in 50-mm culture dishes at a confluence of 5×10^5^ cells/ml. After treatments, total RNA (including miRNAs) was extracted using the miRNeasy kit (Qiagen®), according to the manufacturer’s instructions.

Total RNA extracted from patients, tumour xenografts and cultured cells were quantified using the NanoDrop™ 1000 Spectrophotometer (Thermo Scientific) and the quality was analysed by 2100 Bioanalyzer (Agilent Technologies).

### RT-qPCR analysis

For mRNA quantification, reverse transcription of 500 ng of total RNA was performed using QuantiTect^®^ Reverse Transcription kit (Qiagen^®^), according to the manufacturer’s instructions. TRIM8 and p21 expression levels were measured in triplicate by using TaqMan^®^ assays. Expression levels were calculated relative to the expression levels of RPL13 gene, according to the ∆∆Ct method.

For miRNAs quantification, reverse transcription of 10 ng of total RNA was performed using TaqMan MicroRNA RT Kit (Life Technologies™), according to the manufacturer’s instructions. miR-17-5p, miR-106b-5p, miR-106a-5p, and miR-34a expression levels were measured in triplicate by TaqMan MicroRNA Assay (Life Technologies™) using the ABI PRISM 7900HT platform (Applied Biosystems^®^, Life Technologies™) and normalized to snU6 expression (Life Technologies™).

The reported data represent the average of at least three independent experiments and are shown with their standard deviations or errors. Two-tailed Student’s T tests were performed to assess the statistical significance of gene expression levels differences observed. In this study, a *p*-value less than 0.05 was considered to be statistically significant.

Correlation analysis of TRIM8 and miR-17-5p/miR-106b-5p expression levels was performed using GraphPad Prism 7 (GraphPad Software, San Diego, CA). First, expression data (expressed as ΔCt = Ct_GOI_ - Ct_HKG(s)_) were tested for normality of distribution by D’Agostino-Pearson omnibus K2 test and Shapiro-Wilk test. Then, we computed Pearson correlation coefficients or nonparametric Spearman correlation (with *p*-values) depending on the deviation from Gaussian distribution. Linear regression plots were generated as scatter plots with a trendline by Excel (Microsoft).

### Protein extraction from cell lines and Western blot analysis

Cells were plated in 100-mm culture dishes at a density of 5×10^5^ cells/ml. After treatments, cells were lysed and proteins were extracted as previously described [24].

Tumour masses from xenografts were homogenated in RIPA buffer plus proteinase inhibitors by Tissue Lyser II (25 Hz; 2 min) while tissue samples from patients were homogenated in ice-cold sample buffer (8 M urea, 4% CHAPS, 40 mM Tris-base, 65 mM DTT containing a protease inhibitor cocktail). After 30 min incubation on ice, samples were centrifuged at 13,000 x g at 4 °C. The surnatants were collected in new tubes and protein content was assayed by Bradford dye-binding method (BioRad Protein assay).

For immunoblotting, the following primary antibodies were used: p53 specific DO-1 (Santa Cruz, California, USA 1:300), TRIM8 specific C-20 (Santa Cruz, California, USA 1:200), N-MYC specific GTX81475 (Genetex 1:100), p21 specific C-19 (Santa Cruz, 1:200), PPP2R2B specific (Santa Cruz, California, USA 1:100) and Anti-Actin Ab-1 antibodies kit (Calbiochem, 1:2000). Bound primary antibodies were visualized using Pierce ECL Western Blotting^®^ on a UVITEC Alliance LD2 Cambridge Camera.

The densitometry analysis has been performed using “ImageJ”, a Java-based image-processing program. We used the actin to normalize the data; the protein fold-change has been calculated comparing the values of cells transfected with anti-miR-17-5p or anti-miR-106b-5p to the value of control cells transfected with control miRNA.

## Results

### miR-17-5p and miR-106b-5p expression levels are up-regulated in ccRCC patients

In our previous paper, we demonstrated that TRIM8 is down regulated in ccRCC patients, impairing p53-mediated responses and resulting in chemo-resistance [7]. Here, to elucidate the molecular mechanisms leading to this gene expression alteration, we tested the possible correlation with the expression levels of two miRNA, miR-17-5p and miR-106b-5p, since they are up-regulated in chemo/radio-resistant cancers and the miR-17-92 cluster targets TRIM8 mRNA [8, 9, 13–15].

**Fig. 1:**
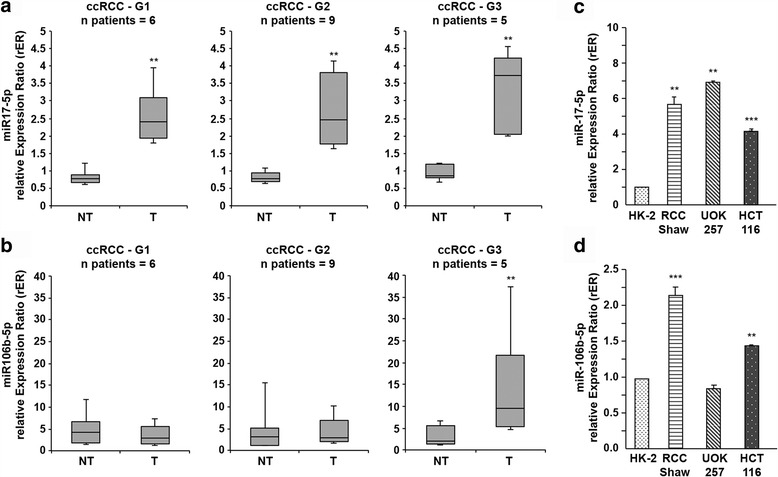
miR-17-5p and miR-106b-5p expression in ccRCC samples. **a**, **b** miR-17-5p and miR-106b-5p expression in ccRCC samples and their paired non-tumour tissues. The analysis was performed considering the Fuhrman grading of the tumour samples. Data are represented in box-and-whisker plots showing median and 10th, 25th, 75th and 90th percentiles for each category of sample. Expression data were measured respect to one normal sample chosen arbitrarily as calibrator and then normalized by the expression levels of U6 snRNA. **p-value < 0.005. **c**, **d** miR-17-5p and miR-106b-5p expression in the colon cancer HCT116 (p53wt) cell line and in three different renal cell lines: the human proximal tubular epithelial cells HK-2, the human renal cell carcinoma RCC-Shaw (p53wt) and the human renal carcinoma of BHD (Birt-Hogg-Dubè) origin UOK-257 cells (mutated-p53). Expression data were measured respect to HK-2 sample chosen as calibrator, and normalized by the expression levels of U6 snRNA. **p-value < 0.005; *** *p*-value < 0.001

We first measured miR-17-5p and miR-106b-5p expression levels in the same ccRCC samples in which we found TRIM8 down-regulation (Fig. 1a and b – Additional file 2: Figure S1a; [7]). The expression levels of miR-17-5p were significantly higher in tumours (T in Fig. 1a), compared to their corresponding non-tumour tissue (NT in Fig. 1a) (3.64-fold; *p*-value < 0.005), which held true for all the sample pairs analysed. Considering the variation of miR-17-5p expression in each tissue pair and Fuhrman grade, no relevant variation was detected suggesting that the up-regulation of miR-17-5p expression might be independent from the severity of this type of tumour (Fig. 1a). On the contrary, miR-106b-5p expression level appeared to be dependent on the tumour grade, since it got significantly higher only in G3 Fuhrman grade samples (Fig. 1b). When miR-17-5p and miR-106b-5p expression levels were compared to those of TRIM8 in the same sample, only miR-17-5p showed a strong negative linear relationship with TRIM8 (r = -0.7878; *p*-value < 0.0001), while miR-106b-5p was not linear correlated to its target gene expression (r = -0.02684; *p*-value = 0.8694) (Additional file 2: Figure S1c and d).

Also in colorectal HCT116 cells and ccRCC derived cell line (RCC-Shaw) the expression levels of miR-17-5p and miR-106b-5p were down-regulated compared to normal human kidney HK-2 cells, in conjunction with an increase of TRIM8 mRNA levels (Fig. 1c and d - Additional file 2: Figure S1b). Intriguingly, the UOK-257 carcinoma cell line (harbouring a mutant p53) showed miR-17-5p expression levels comparable to cancer RCC-Shaw and HCT116 cells, while miR-106b-5p expression levels were comparable to non-cancer HK-2 cells (Fig. 1c and d), a condition similar to that observed in G1-G2 Fuhrman grade patients (where p53 was wild-type though).

Significantly, ccRCC tissues and their non-tumour counterpart showed no differences at all in the expression levels of miR-106a-5p, belonging to the same miR-17 “seed” family and whose “seed” region matched with TRIM8-3’UTR (Additional file 3: Figure S2a).

Interestingly, we did not observe alterations of miR-106a-5p, miR-17-5p and miR-106b-5p expression in benign renal oncocytoma samples compared to non-tumour matched epithelial tissues (Additional file 3: Figure S2b), suggesting that only miR-17-5p and miR-106b-5p up-regulation is a signature of a malignant phenotype.

Altogether, these results clearly indicate a strong correlation between TRIM8 mRNA expression and miR-17-5p and miR-106b-5p levels, suggesting that these miRNAs could mediate TRIM8 mRNA degradation.

### TRIM8 3’UTR is a target of both miR-17-5p and miR-106b-5p

**Fig. 2:**
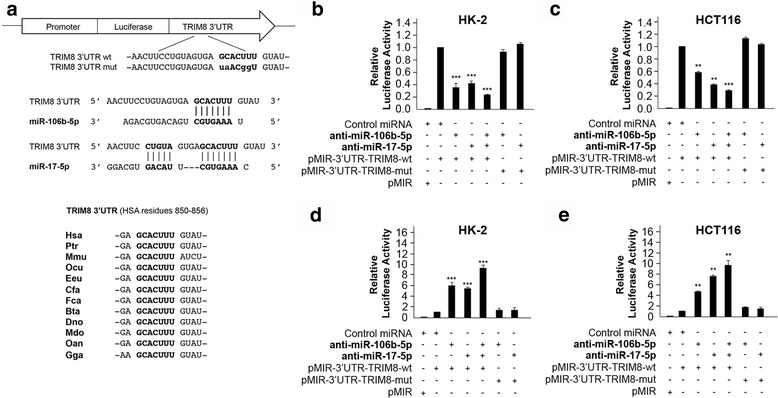
Structure and functional characterization of the putative miR-17-5p/miR-106b-5p target identified in the TRIM8 3’UTR sequence. **a** Schematic representation of the pMIR luciferase reporter construct containing the TRIM8 3’UTR sequence (wild-type or mutated) cloned downstream the Luciferase gene. Below it is shown the sequence alignment between the miR-17-5p/miR-106b-5p “seed” sequence and the TRIM8 3’UTR, as well as the evolutionary conservation across species. **b**, **c**, **d**, **e** Luciferase assays. The HK-2 and HCT116 cells were transfected with Negative Control miRNA Mimic, miR-17-5p or miR-106b-5p (alone or together), anti-miR-17-5p or anti-miR-106b-5p (alone or together), along with pMIR luciferase reporter construct containing TRIM8 3’UTR (wt or mut). Cells were lysed and luciferase activity was determined as described in the Material and Methods section. Transfection efficacy was normalized by Renilla Luciferase activity. Data represent the averages of at least three independent experiments with their standard deviations. ** *p*-value < 0.005; *** *p*-value < 0.001

To demonstrate that miR-106b-5p as well as miR-17-5p directly inhibit the expression of TRIM8, we first used an *in silico* approach to identify the miR-106b-5p and miR-17-5p-binding sequence in the TRIM8 3’UTR region by using Target Scan (Release7.0, August 2015) [25], the database of conserved 3’UTR miRNA targets. We found that both miRNAs seed regions perfectly matched an evolutionarily conserved region in the 3’UTR of the TRIM8 mRNA (Fig. 2a), which we experimentally tested by performing Luciferase Reporter assay. We cloned the putative binding sites (wild-type or suitably mutated) of miR-106b-5p and miR-17-5p downstream of the *luc2* firefly luciferase gene, under the control of the human PhosphoGlycerateKinase (PGK) promoter (pMIR-3’UTR-TRIM8-wt or pMIR-3’UTR-TRIM8-mut) and transfected them in the HK-2 and HCT116 cell lines with Negative Control miRNA Mimic (Ambion), miR-106b-5p, miR-17-5p, anti-miR-106b-5p, anti-miR-17-5p, both miRNAs or both anti-miRNAs (Fig. 2b-e). The efficiency of the transfections was validated by RT-qPCR (data not shown). The luciferase reporter assays demonstrated that both miR-106b-5p and miR-17-5p significantly suppressed the firefly luciferase activity of pMIR-3’UTR-TRIM8-wt (2.63- and 2.44-fold in HK-2, 1.82- and 2.6-fold in HCT116, respectively), whereas they failed to work when the target site was mutated (Fig. 2b and c). The co-transfection of both miR-106b-5p and miR-17-5p further decreased the luciferase activity (4.2-fold in HK-2 and 3.56-fold in HCT116 cells) (Fig. 2b and c), indicating they may act synergistically. On the other side, the inhibition of both endogenous miR-106b-5p and miR-17-5p by anti-miR-106b-5p and anti-miR-17-5p resulted in increasing firefly luciferase activity of pMIR-3’UTR-TRIM8-wt, unlike the mutant construct (Fig. 2d and e).

As p21 mRNA contains a binding site for miR-106b-5p and miR-17-5p [26, 27], we cloned its 3’UTR in the pMIR luciferase vector (pMIR-3’UTR-p21-wt) and used it to gauge the specificity of both miRNAs activity (Additional file 4: Figure S3a and b).

**Fig. 3:**
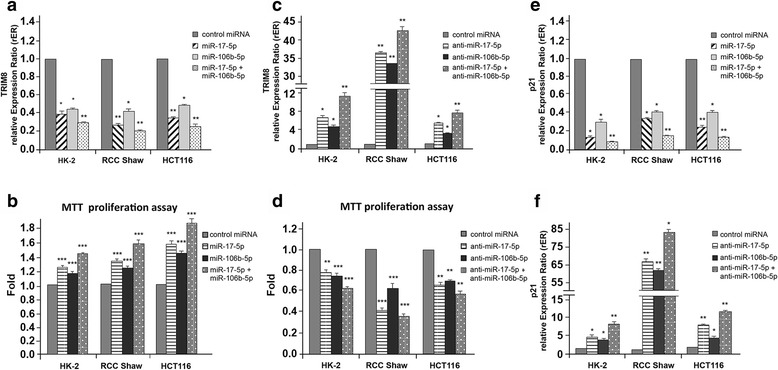
Effects of overexpression/silencing of miR-17-5p and miR-106b-5p on TRIM8 and p21 expression and cell proliferation. **a**, **c**, **e**, **f** Expression levels of TRIM8 and p21 were measured by RT-qPCR in HK-2, RCC-Shaw and HCT116 cells transfected with Negative Control miRNA Mimic, miR-17-5p or miR-106b-5p (alone or together) anti-miR-17-5p or anti-miR-106b-5p (alone or together). Relative expression ratios were measured respect to the sample transfected with the Negative Control miRNA Mimic and normalized by the expression levels of RPL13 (* *p*-value < 0.01; ** *p*-value < 0.005). **b**, **d** Cell proliferation was measured by MTT reduction assay in HK-2, RCC-Shaw and HCT116 transfected with Negative Control miRNA Mimic, miR-17-5p or miR-106b-5p (alone or together), anti-miR-17-5p or anti-miR-106b-5p (alone or together). Measurements were normalized respect to the sample transfected with the Negative Control miRNA Mimic. Data are shown as the average with standard deviation of at least 3 independent experiments (** *p*-value < 0.005; *** *p*-value < 0.001)

To further confirm our hypothesis, we analysed the effect of miR-106b-5p or miR-17-5p overexpression or repression by transient transfection of the specific anti-miR-106b-5p and anti-miR-17-5p in HK-2, clear cell Renal Carcinoma (RCC-Shaw) and colorectal HCT116 cell lines. RT-qPCR demonstrated that the overexpression of both miRNAs decreased TRIM8 mRNA steady state levels and, coherently, increased cell proliferation (Fig. 3a and b). On the contrary, the suppression of miR-106b-5p or miR-17-5p by specific anti-miRNAs increased TRIM8 expression levels with a reduction of cell proliferation in all cell lines (Fig. 3c and d). The simultaneous expression of both miRNAs caused a further decrease of TRIM8 mRNA levels (Fig. 3a), while their simultaneous suppression further promoted TRIM8 expression (Fig. 3c). Similar results were obtained for p21 mRNA control target (Fig. 3e and f).

Altogether, our experiments demonstrated that TRIM8 is a direct target of miR-106b-5p as well as of miR-17-5p and that their co-expression synergizes in decreasing TRIM8 mRNA, eventually resulting in increased cell proliferation (Fig. 3a-d).

### Both miR-17-5p and miR-106b-5p link p53 to the N-MYC pathway

**Fig. 4:**
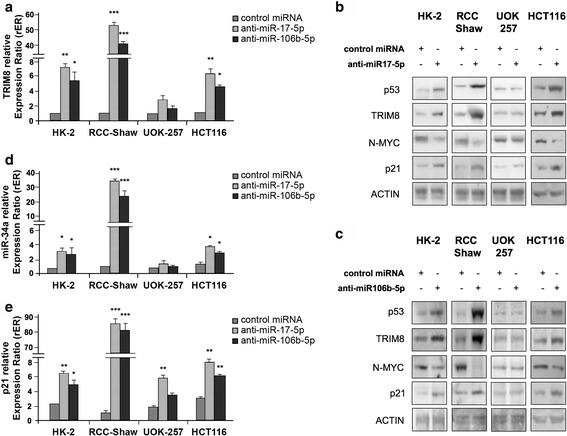
miR-17-5p and miR-106b-5p link p53 to the N-MYC pathway. **a**, **d**, **e** Expression levels of TRIM8, p21 and miR-34a were measured by RT-qPCR in HK-2, RCC-Shaw, UOK-257 and HCT116 transfected with Negative Control miRNA Mimic, anti-miR-17-5p or anti-miR-106b-5p. Relative expression ratios were measured respect to the sample transfected with the Negative Control miRNA Mimic and normalized by the expression levels of RPL13 for TRIM8 and p21, and by the expression level of U6 snRNA for miR-34a (** *p*-value < 0.005; *** *p*-value < 0.001). **b**, **c** Western blotting analysis of p53, TRIM8, N-MYC and p21 proteins were measured in the indicated cell lines transfected with Negative Control miRNA Mimic, anti-miR-17-5p or anti-miR-106b-5p. Western blot of Actin was conducted as control. Data are shown as the average with standard deviation of at least 3 independent experiments (** *p*-value < 0.005; *** *p*-value < 0.001)

The increase of miR-17-5p and of miR-106b-5p (such as that we observed in G3 stage renal carcinomas, Fig. 1a and b) might account for a TRIM8 deficit, possibly responsible for p53 inactivation even in the absence of p53 mutations. Indeed, miR-17-92 cluster and MCM7 gene, this latter containing the miR-106b-25 cluster, are transcriptionally activated by N-MYC, which in turn is negatively regulated by miR-34a, whose expression is regulated by p53 [17–20]. We hence tested if the inhibition of these two miRNAs by specific anti-miRNAs would lead to p53 reactivation and to p53-dependent N-MYC destabilization through miR-34a up-regulation [17, 20]. The HK-2 (normal kidney cells), the RCC-Shaw (ccRCC-derived cell line with wt-p53), the UOK-257 cells (RCC cell line with mutated p53) and HCT116 (colorectal cancer with wt-p53) were transfected with anti-miR-17-5p and anti-miR-106b-5p. The efficiency of the anti-miRNAs transfections was validated by RT-qPCR (Additional file 5: Figure S4a and b). HK-2, RCC-Shaw and HCT116 cells, but not p53-mutant UOK-257 cells, showed a relevant increase of TRIM8 mRNA and protein levels (Fig. 4a-c and Additional file 6: Figure S5a and b). Accordingly, only in p53wt cell lines, i.e. HK-2, RCC-Shaw and HCT116, p53 became stabilized, miR-34a and p21 levels increased, while N-MYC protein levels sensibly dropped (Fig. 4b-e and Additional file 6: Figure S5a and b). In UOK-257 cells, upon anti-miR-17-5p overexpression, p21 protein expression increased 2.3 fold compared to the control cells coherently with mRNA expression (Fig. 4b and e - Additional file 6: Figure S5a). This increase in p21 expression seems to be p53-independent since we did not observe any increase of p53 protein upon anti-miR-17-5p overexpression (Adiitional file 6: Figure S5a).

As a final important result of miR-17-5p and miR-106b-5p knocking-down, RCC and HCT116 cell proliferation rate decreased as demonstrated by MTT and colony-forming assays (Fig. 3d - Additional file 5: Figure S4c).

**Fig. 5:**
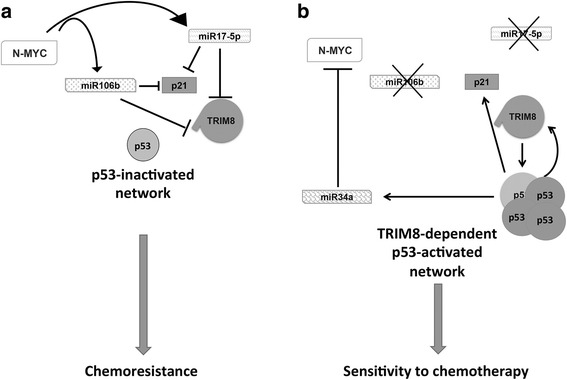
Schematic representation of the opposite p53-TRIM8 and N-MYC-miR17-5p/miR106b-5p networks in cellular response to treatments. **a**, **b** Schematic representation of the molecular pathway that shows how TRIM8 recovery in TRIM8-deficient cells reactivates the p53 tumour suppressor pathway and blunts the N-MYC oncogenic activity

These results clearly depict a novel pathway through which the overexpression of miR-17-5p and its paralogue miR-106b-5p inhibits TRIM8, leading, on one hand, to the de-stabilization of the p53 tumour suppressor protein and, on the other hand, to the activation of the N-MYC oncogene, turning on cancer cells proliferation (Fig. 5a). A feed-forward loop is thus created, whereby N-MYC fosters its own protein expression via the inhibition of TRIM8 mRNA by promoting miR-17-5p and miR-106b-5p expression (Fig. 5a). Conversely, by inhibiting the action of miR-17-5p and miR-106b-5p on TRIM8, p53 becomes stable and activates miR-34a expression, which in turn quenches the N-MYC protein (Fig. 5b).

### Anti-miR-17-5p and anti-miR-106b-5p render chemotherapy effective

The low chemotherapy response characterising ccRCC and CRC prompted us to investigate whether miR-17-5p and miR-106b-5p might contribute to the chemotherapy resistance due to the TRIM8 protein loss, in the two paradigmatic models of these cancers.

**Fig. 6:**
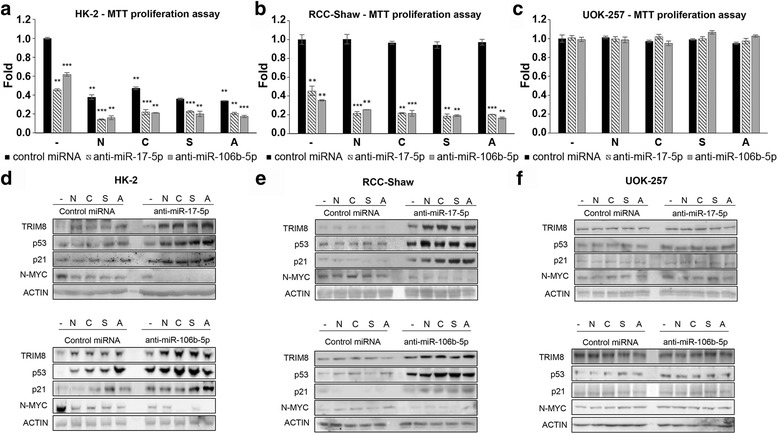
Anti-miR-17-5p and anti-miR-106b-5p render chemotherapy treatments effective in ccRCC. **a**, **b**, **c** Cell proliferation by MTT reduction assay in HK-2 (p53 wt), RCC-Shaw (p53 wt), and UOK-257 (mutated p53) transfected with Negative Control miRNA Mimic, anti-miR-17-5p or anti-miR-106b-5p, and treated with Nutlin-3 (N)(10 μM), Cisplatin (C)(7.5 μM), Sorafenib (S)(10 μM), Axitinib (A)(10 μM) or drug-untreated cells(-). For each cell line, drug-untreated sample transfected with control miRNA has been used as calibrator (fold 1.0). Cells transfected with anti-miR-17-5p or anti-miR-106b-5p, or with control miRNA and treated with the different drugs have been normalized with respect to this calibrator. Data are shown as the average with standard deviation of at least 3 independent experiments (** *p*-value < 0.005; *** *p*-value < 0.001). **d**, **e**, **f** Western Blotting of the indicated proteins in HK-2 (control), RCC-Shaw and UOK-257 after transfection with Negative Control miRNA Mimic, anti-miR-17-5p, anti-miR-106b-5p without drug (-) or after chemotherapeutic drug treatment with Nutlin-3 (N)(10 μM), Cisplatin (C)(7.5 μM), Sorafenib (S)(10 μM) or Axitinib (A)(10 μM). Western blot of Actin was conducted as control

To address this critical question, renal HK-2, RCC-Shaw and UOK-257 cells were transfected with control miRNA, anti-miR-17-5p or anti-miR-106b-5p and treated with Nutlin-3 (N) and Cisplatin (C), which induce p53-dependent cell cycle arrest in tumours with wild type p53. Moreover we treated the cells also with Sorafenib (S) or Axitinib (A), belonging to the class of tyrosine kinases inhibitors (TKIs) that are currently the most successful class of drugs used in the treatment of renal, hepatocellular carcinoma, colorectal and thyroid cancer in adults [28–36]. The efficiency of the anti-miRNAs transfections was validated by RT-qPCR (Additional file 7: Figure S6a-f). MTT proliferation and colony suppression assays demonstrated that all the chemotherapeutic drugs used significantly reduced HK-2 cell proliferation rate, but had no effect at all on both RCC-Shaw and UOK-257, which have in common inactivated p53, the first due to TRIM8 deficit, the second to a mutation in the *TP53* gene itself (Fig. 6a-c - Additional file 7: Figure S6g-i). Conversely, both anti-miR-17-5p and anti-miR-106b-5p induced a significant reduction in proliferation rate in RCC-Shaw and in HK-2 cells, but not in UOK-257 cells, which became more pronounced when cells were treated with chemotherapeutics (Fig. 6a-c).

Noteworthy, RT-qPCR demonstrated that in RCC-Shaw cells all the drugs induced a great increase of miR-17-5p and weaker of miR-106b-5p expression levels (Additional file 7: Figure S6b and e). This effect would further repress TRIM8 and p21, contributing positively to the growth of the tumour and to the onset of chemo-resistance (Fig. 6a-c - Additional file 7: Figure S6g-i).

Consistently with the cell proliferation decrease upon anti-miR-17-5p and anti-miR-106b-5p overexpression, only in p53wt background, but not in p53-mutated UOK-257 cells, TRIM8 protein levels raised, p53 became stabilized and promoted p21 and miR-34a transcription, in turn decreasing N-MYC protein levels (Fig. 6d-f and Additional file 8: Figure S7a-i).

**Fig. 7:**
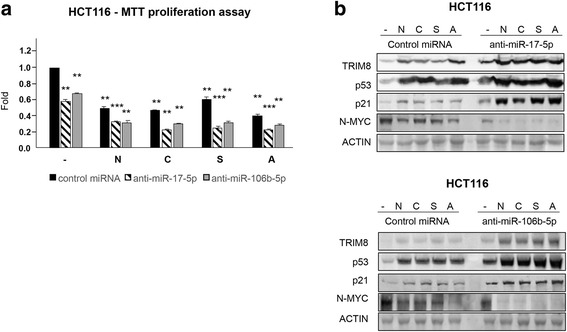
Anti-miR-17-5p and anti-miR-106b-5p render chemotherapy treatments effective in CRC. **a** Cell proliferation by MTT reduction assay in HCT116 (p53 wt) cells transfected with Negative Control miRNA Mimic, anti-miR-17-5p or anti-miR-106b-5p, and treated with Nutlin-3 (N)(10 μM), Cisplatin (C)(7.5 μM), Sorafenib (S)(10 μM), Axitinib (A)(10 μM) or drug-untreated cells(-). For each cell line, drug-untreated sample transfected with control miRNA has been used as calibrator (fold 1.0). Cells transfected with anti-miR-17-5p or anti-miR-106b-5p, or with control miRNA and treated with the different drugs have been normalized with respect to this calibrator. Data are shown as the average with standard deviation of at least 3 independent experiments (** *p*-value < 0.005; *** *p*-value < 0.001). **b** Western Blotting of the indicated proteins in HCT116 cells after transfection with Negative Control miRNA Mimic, anti-miR-17-5p, anti-miR-106b-5p without drug (-) or after chemotherapeutic drug treatment with Nutlin-3 (N)(10 μM), Cisplatin (C)(7.5 μM), Sorafenib (S)(10 μM) or Axitinib (A)(10 μM). Western blot of Actin was conducted as control

As stated before, colorectal HCT116 cells show higher expression level of TRIM8 and coherently lower miR-17-5p and miR-106b-5p expression levels than RCC-Shaw cells (Fig. 1c and d - Additional file 2: Figure S1b). Even though chemotherapeutic drugs are able to reduce cell proliferation rate, this reduction became more pronounced when we increased TRIM8 and p53 proteins levels by transfecting anti-miR-17-5p or anti-miR-106b-5p (Fig. 7a-b), leading to miR-34a up-regulation and N-MYC decrease (Fig. 7b - Additional file 9: Figure S8a-f).

Altogether, these experiments demonstrated that anti-miR-17-5p and anti-miR-106b-5p increase the efficacy of chemotherapy treatments in resistant cancer cell lines.

**Fig. 8:**
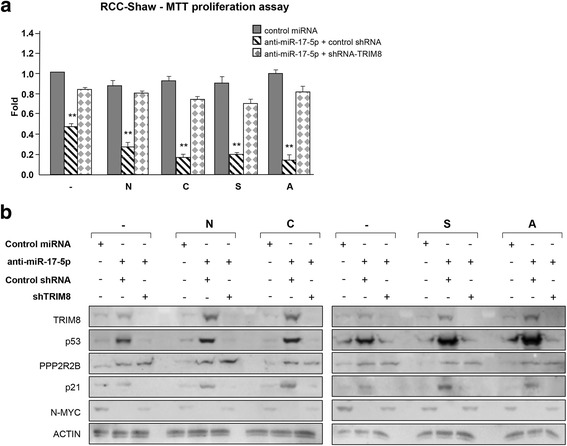
TRIM8 is pivotal in controlling chemotherapy cell sensitivity. **a** Cell proliferation by MTT reduction assay in RCC-Shaw transfected with Negative Control miRNA Mimic or anti-miR-17-5p plus control short hairpin-RNA (control shRNA) or specific short hairpin against TRIM8 (shRNA-TRIM8) (** *p*-value < 0.005). After transfection the cells were treated with Nutlin-3 (N) (10 μM), Cisplatin (C) (7.5 μM), Sorafenib (S) (10 μM), Axitinib (A) (10 μM) or drug-untreated cells(-). For each cell line, drug-untreated sample transfected with control miRNA has been used as calibrator (fold 1.0). Cells transfected with anti-miR-17-5p plus control shRNA or shRNA-TRIM8, or with control miRNA and treated with the different drugs have been normalized with respect to this calibrator. **b** Western blotting analysis of the indicated proteins in RCC-Shaw transfected with Negative Control miRNA Mimic or anti-miR-17-5p plus control short hairpin-RNA or specific short hairpin against TRIM8 (shRNA-TRIM8). After transfection the cells were treated with Nutlin-3 (N) (10 μM), Cisplatin (C) (7.5 μM), Sorafenib (S) (10 μM), Axitinib (A) (10 μM) or drug-untreated cells (-). Western blot of Actin was conducted as control

Next, we tested if TRIM8, among all the targets regulated by miR-17-5p, was pivotal to trigger cell sensitivity to chemotherapy. To tackle this issue, RCC-Shaw cells were transfected with Negative Control miRNA Mimic (Ambion), anti-miR-17-5p plus pRS control vector or anti-miR-17-5p plus specific TRIM8 short hairpins (pRS-shRNA-TRIM8) and treated with chemotherapeutics. As shown in Fig. 8a-b and in Additional file 10: Figure 9a-d, anti-miR-17-5p plus control shRNA blocked cell proliferation because TRIM8 and p53 expression levels were increased, p21 and miR-34a were transactivated by p53, and N-MYC was destabilized. On the contrary, anti-miR-17-5p plus specific TRIM8 short hairpin failed to stall cell proliferation because p53 was not stabilized by TRIM8, and was therefore no longer able to transactivate p21 and miR-34a (Fig. 8b - Additional file 10: Figure S9a-d). Accordingly, N-MYC was still present (Fig. 8b). The successful neutralization of miR-17-5p by anti-miR-17-5p was demonstrated by the stabilization of one of its targets, i.e. PPP2R2B (Protein Phosphatase 2 Regulatory subunit B, beta) (Fig. 8b) [37].

**Fig. 9:**
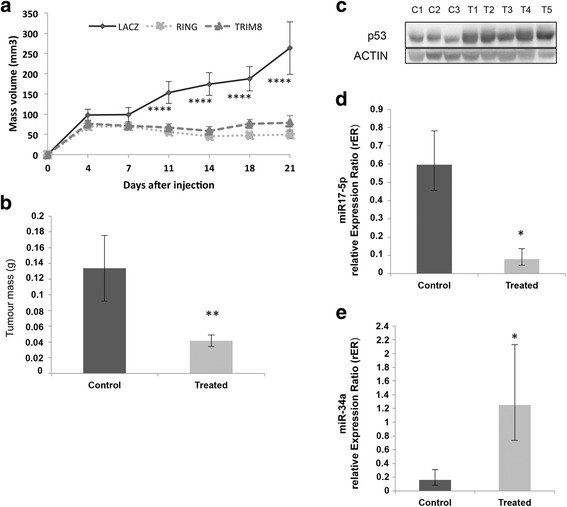
Treatment of xenograft tumours in vivo with recombinant adenovirus expressing HA-TRIM8, HA-RING or LacZ (Control). **a** Growth curves of xenograft tumours in nude mice treated for 3 weeks. The volume of the tumours was measured two times weekly (**** *p*- value <0.0005). **b** Tumour masses weight at the moment of the tumour excision in treated (HA-TRIM8, HA-RING) vs. control (LacZ) samples (** *p*-value < 0.005). **c**, **d**, **e** The stabilization of p53, miR-34a and down-regulation of miR-17-5p in xenografts treated with HA-TRIM8, HA-RING (T1-T5) or LacZ (C1-C3) was demonstrated by western blotting analysis of p53 (Actin was used as loading control) and by RT-qPCR on miR-17-5p (* *p*-value < 0.05) and miR-34a expression (* *p*-value < 0.05). The bars represent the Standard Error of the Mean

These results led to the conclusion that TRIM8, among all miR-17-5p targets, is pivotal in controlling cell sensitivity to chemotherapy. Therefore, the effectiveness of anti-miR-17-5p and anti-miR-106b-5p in rendering the cells sensitive to chemotherapeutic drugs may be explained by their capability to increase TRIM8 mRNA steady state levels.

### TRIM8 overexpression blocks cell proliferation of human tumour xenografts in nude mice

Based on the in vitro experiments, we hypothesized that TRIM8 over-expression might inhibit tumour growth in vivo, and attempted to demonstrate it by injecting human cancer cells into nude mice. We next treated the tumours generated by administering TRIM8 or RING-TRIM8, since we demonstrated that the RING domain of TRIM8 alone is responsible for both p53 stabilization/activation and MDM2 degradation [2]. We did not treat tumours with anti-miR-17-5p because its inhibitory effects on tumour growth have been reported [26], while our aim here was to point out the critical role of TRIM8 on tumour growth. To treat cancer cells with TRIM8 or RING-TRIM8, we devised a strategy based on the use of recombinant replication-incompetent adenoviruses. Hence, three recombinant adenoviruses expressing HA-TRIM8, HA-RING-TRIM8 or LacZ (as control) were generated.

To generate xenografts, we decided to use the metastatic colon cancer HCT116 (p53+/+) cell line, which shows a high tumorigenic potential and whose growth kinetics in nude mice are well known [38]. HCT116 cultured cells were injected subcutaneously in nude mice and tumours thereby generated were treated by each of the three recombinant adenoviruses administered twice a week for three weeks. Eventually, tumours were measured by caliper at the same time.

As shown in Fig. 9a, the xenograft growth curves revealed that the size of the tumours treated with Ad-HA-TRIM8 or Ad-HA-RING remained unchanged and, at the end of the experiment, they displayed mainly the same size they had at the moment of injection. Conversely, the control masses injected with the LacZ recombinant adenovirus grew with an exponential trend (Fig. 9a). The effect of the treatment is evident also in the size and weight of the masses that were markedly smaller than the control ones (Fig. 9b - Additional file 11: Figure S10a). As we observed in vitro, molecular analyses of the excised masses confirmed p53 stabilization following exogenous HA-TRIM8/RING expression, miR-17-5p down-regulation and, at the same time, the up-regulation of miR-34a (Fig. 9c-e - Additional file 11: Figure S10b).

These evidences confirmed in vivo the pathway we identified in vitro, with TRIM8 emerging as key actor of the activation in the p53-(miR-34a)-(N-MYC)-(miR-17) family.

Moreover, these results strongly suggest the crucial role of TRIM8 protein in preventing tumour growth in vivo.

## Discussion

In this paper, we describe for the first time how TRIM8 plays a crucial role in p53 activation and in N-MYC quenching in a complex signalling involving the miR17 family. Moreover, we demonstrate that a TRIM8 deficit, due to miR-17-5p/miR-106b-5p up-regulation, contributes to oncogenesis and chemo-resistance.

We used two paradigmatic models of chemo-resistant cancers: Renal Cell Carcinoma (RCC) and Colorectal Cancer (CRC). RCC is the seventh most common neoplasm in men and the ninth most commonly occurring in women. It is not responsive to neither chemotherapy nor radiation therapy when metastases are already present and is not possible to perform surgery for approximately 30% of all RCC cases [39]. CRC is one of the leading cause of cancer mortality worldwide and shows poor prognosis in advanced forms because of the high rate of resistance to radiotherapy or chemotherapy, which leads to recurrence, metastasis and death [40, 41].

Previously, we demonstrated that TRIM8 is down-regulated in ccRCC, impairing p53-mediated responses to chemotherapeutic drugs [7]. This deficit is partially due to the loss of TRIM8 heterozygosity, but we did not exclude other additional mechanisms, such as epigenetic silencing. Here we report an inverse correlation between miR-17-5p and TRIM8 expression in vivo. In patients with a more aggressive tumour behaviour (G3 Fuhrman grade) we observed also a significant increase of miR-106b-5p, but not miR-106a-5p, belonging both to the same miR-17 seed-family. In patients affected by colorectal cancer, neuroblastoma, breast and pancreatic cancer, it has been extensively reported that miR-17-5p and miR-106b-5p are overexpressed and are capable to confer chemo-resistance [14, 15, 42, 43].

A shared feature between these two miRNAs, in addition to the seed region, is that their transcription is promoted by the oncoprotein N-MYC. Indeed, N-MYC transactivates the miR17-92 cluster and may be considered a bona fide activator of the miR-106b-25 cluster, since the *MCM7* gene (mini-chromosome maintenance) is transactivated by N-MYC [18, 19]. Of note, the miR-106b/25 cluster is located exactly within intron 13 of the *MCM7* gene. MCM7 protein possesses the vital function of “licensing” DNA synthesis during the transition from G1 to the S phase. Both miRNAs clusters, in turn, down-modulate different targets, beside TRIM8, such as the tumour suppressors p21 and PTEN, hence contributing to tumorigenesis [14, 15]. Nevertheless, among all the targets regulated by miR-17-5p, we demonstrated that TRIM8 is pivotal to trigger cell sensitivity to chemotherapy.

In mammalian cells, MYC proteins belong to three distinct gene families of transcription factors, namely C-MYC, L-MYC and N-MYC. Their powerful oncogenic fame derives from their frequent deregulation in a myriad of human cancers and from a series of activities that place MYC at the nexus of cell growth, proliferation, metabolism, and genome stability [16]. MYC proteins are subject to stringent control at every step of their expression. There are several potential ways by which cancer cells deregulate MYC, leading all to overexpression of MYC proteins and their disconnection from the critical signalling processes that normally keep it in check. C-MYC is overexpressed in many different cancers; L-MYC is most often overexpressed in small cell lung carcinomas, while N-MYC is most frequently overexpressed in solid cancers of neural origin, as neuroblastoma and glioma. Only very few studies reported the up-regulation of C-MYC pathway in ccRCC, even without MYC amplification [44]. Notably, no detailed studies on the biological and clinical significance of N-MYC have been reported for ccRCC and CRC thus far, to the best of our knowledge.

Here we showed that the up-regulation of miR-17-5p and miR-106b-5p leads to TRIM8 deficit, which in turn leads to failure of p53 protein activation, preventing the cells response to chemotherapy. Surprisingly, RT-qPCR demonstrated that RCC cells treated with chemotherapeutic drugs showed an inexplicable increase of miR-17-5p and miR-106b-5p expression levels (Additional file 7: Figure S6b and e) that in addition to keeping low TRIM8 protein levels, also keeps low the levels of crucial cell cycle inhibitors such as p21 and PTEN [14, 15].

Previously, we observed that in ccRCC cell lines, differently from other cell lines, TRIM8 do not promote degradation of MDM2, the main negative regulator of p53 [7]. With respect to this, it was demonstrated that N-MYC up-regulates baseline levels of MDM2, inhibiting p53-triggered apoptosis in neuroblastoma [45]. In this scenario, TRIM8 recovery via miR-17-5p and miR-106b-5p silencing seems to be a winning move as it renders effective the tumour suppressor activity of p53, promoting the transcription of miR-34a that knocks out the oncogenic potential of N-MYC. Therefore we demonstrated that, by counteracting the action of miR-17-5p and miR-106b-5p by specific anti-miRs, the cells regained sensitivity to chemotherapeutic treatments. Significantly, cells became sensitive not only to Sorafenib and Axitinib, which are among Tyrosine Kinase Inhibitors currently in use for treatment of both renal and colorectal carcinoma [28, 30, 32, 34, 35], but also to Nutlin-3 and Cisplatin. This suggests that with the TRIM8-mediated recovery of the p53 tumour suppressor activity, a broader spectrum of chemotherapeutic agents may be taken into consideration to blunt tumorigenicity. Indeed, it is known that Nutlin-3 enhances the efficacy of Sorafenib in renal cell carcinoma, pointing to synergistic effects of these two drugs in the treatment of this cancer [21].

Finally, the suppression of tumour cell proliferation in mice xenografts provides conclusive evidence of the TRIM8 role in mediating the p53 response in tumours.

## Conclusions

In this paper, we report that low expression levels of TRIM8 are an adverse prognostic factor. Our studies increase the understanding of the molecular mechanisms underlying the malignant features of chemo-resistant tumours, suggesting molecular criteria for selecting targeted therapies. The paradigm for cancer treatment has evolved from relatively non-specific cytotoxic agents to selective, mechanism-based therapeutics. Therefore, understanding how cancer cells lose control of MYC expression and p53 tumour suppressor activities is immensely important in terms of staging cancers, predicting outcomes to designed therapies.

Potential therapeutic applications that target miR-17-5p/miR-106b-5p using specific anti-miRNAs should be considered more closely for chemo-resistant tumours.

## Additional files

Additional file 1: Table S1. Main characteristics of patients affected by clear cell Renal Cell Carcinoma (ccRCC) and renal oncocytoma (RO). The anagraphic (gender and age) and clinical (Fuhrman grade) data are reported. (XLSX 65 kb)
Additional file 2: Figure S1. (a) TRIM8 expression in ccRCC samples (T) and their paired non-tumour tissues (NT). The analysis was performed considering the Fuhrman grading of the tumour samples. Data are represented in box-and-whisker plots showing median and 10^th^, 25^th^, 75^th^ and 90^th^ percentiles for each category of sample. Expression data were measured respect to one normal sample chosen arbitrarily as calibrator and then normalized by the geometric mean of RPL13 and ACTB relative expression ratios. The bars represent the Standard Error of the Mean. (b) TRIM8 expression in three different renal cell lines: the human proximal tubular epithelial cells HK-2, the human renal cell carcinoma RCC-Shaw (p53wt) and the human renal carcinoma of BHD (Birt-Hogg-Dubè) origin UOK-257 cells (mutated-p53) and the colon cancer HCT116 (p53wt) cell line. Expression data were measured respect to HK-2 sample chosen as calibrator, and normalized by the expression levels of RPL13. ***p*-value < 0.005; *** *p*-value < 0.001. (c, d) Linear regression plots of miR-17-5p and miR-106b-5p expression levels compared with TRIM8 expression levels in ccRCC samples. On the x-axis the Ct of TRIM8 minus the arithmetic mean of Cts calculated for ACTB and RPL13 (used as housekeeping genes in RT-qPCR analysis) is reported, while the y-axis shows the Ct of miR-17-5p/miR-106b-5p minus the Ct of U6 snRNP (used to normalize miRNAs expression levels). The value of the correlation coefficient “r” and the *p*-value are indicated on the graph. Dots are coloured differentially according to the sample type: grey for non-tumour samples, green for Fuhrman Grade 1 samples, orange for Fuhrman Grade 2 samples and yellow for Fuhrman Grade 3 samples. (PDF 186 kb)
Additional File 3: Figure S2. (a) miR-106a-5p expression in ccRCC samples (T) and their paired non-tumour tissues (NT). The analysis was performed considering the Fuhrman grading of the tumour samples. Data are represented in box-and-whisker plots showing median and 10^th^, 25^th^, 75^th^ and 90^th^ percentiles for each category of sample. Expression data were measured respect to one normal sample chosen arbitrarily as calibrator and then normalized by the expression levels of U6 snRNA. The bars represent the Standard Error of the Mean. It is also reported the sequence alignment between the miR-106a-5p “seed” sequence and the TRIM8 3’UTR. (b) miR-106a-5p, miR-17-5p and miR-106b-5p expression in 4 oncocytoma samples (T) and their paired non-tumour tissues (NT). Data are represented in box-and-whisker plots showing median and 10th, 25th, 75th and 90th percentiles for each category of sample. Expression data were measured respect to one normal sample chosen arbitrarily as calibrator and then normalized by the expression levels of U6 snRNA. The bars represent the Standard Error of the Mean. (PDF 79 kb)
Additional file 4: Figure S3. (a, b) Luciferase assay. The HK-2 and the HCT116 cells were transfected with Negative Control miRNA Mimic, miR-17-5p, miR-106b-5p, anti-miR-17-5p or anti-miR-106b-5p, along with pMIR luciferase reporter construct containing p21 3’UTR. Cells were lysed and luciferase activity was determined as described in the Material and Methods section. Transfection efficacy was normalized by Renilla Luciferase activity. Data represent the averages of at least three independent experiments with their standard deviations. ** *p*-value < 0.005; *** *p*-value < 0.001. (PDF 64 kb)
Additional file 5: Figure S4. (a, b) Expression levels of miR-17-5p and miR-106b-5p were measured by qPCR in HK-2, RCC-Shaw, UOK-257 and HCT116, transfected with Negative Control miRNA Mimic, anti-miR-17-5p or anti-miR-106b-5p. Relative expression ratios were measured respect to the sample transfected with the Negative Control miRNA Mimic and normalized by the expression level of U6 snRNA. The bars represent the Standard deviation of the Mean. (c) Colony suppression assays. Cell growth was measured in HK-2, RCC-Shaw, UOK-257 and HCT116 cells transfected with Negative Control miRNA Mimic, anti-miR-17-5p or anti-miR-106b-5p. (PDF 1350 kb)
Additional file 6: Figure S5. (a-b) Densitometry analysis of p53, Trim8, N-MYC and p21 proteins in HK-2, UOK-257, RCC-Shaw and HCT116 cells transfected with negative control miRNA Mimic, anti-miR-17-5p (a) or anti-miR-106b-5p (b). The relative amounts of proteins, reported in the table below the western blotting, have been calculated normalizing to actin and calibrating to the respective control. (PDF 925 kb)
Additional file 7: Figure S6. (a-f) Expression levels of miR-17-5p and miR-106b-5p were measured by RT-qPCR in HK-2, RCC-Shaw and UOK-257 cells, transfected with Negative Control miRNA Mimic, anti-miR-17-5p or anti-miR-106b-5p, and treated for 24 h with Nutlin-3 (N) (10 μM), Cisplatin (C) (7.5 μM), Sorafenib (S) (10 μM), Axitinib (A) (10 μM) or drug-untreated cells (-). Relative expression ratios were measured respect to the sample transfected with the Negative Control miRNA Mimic and normalized by the expression level of U6 snRNA. ** *p*-value < 0.005 (g-i) Colony suppression assay. Cell growth were measured in HK-2, RCC-Shaw and UOK-257 cells transfected with Negative Control miRNA Mimic, anti-miR-17-5p or anti-miR-106b-5p, and treated for 24 h with Nutlin-3 (N) (10 μM), Cisplatin (C) (7.5 μM), Sorafenib (S) (10 μM), Axitinib (A) (10 μM) or drug-untreated cells (-). (PDF 380 kb)
Additional file 8: Figure S7. (a-i) Expression levels of TRIM8, p21 and miR-34a were measured by RT-qPCR in HK-2, RCC-Shaw and UOK-257 cells, transfected with Negative Control miRNA Mimic, anti-miR-17-5p or anti-miR-106b-5p, and treated for 24 h with Nutlin-3 (N) (10 μM), Cisplatin (C) (7.5 μM), Sorafenib (S) (10 μM), Axitinib (A) (10 μM) or drug-untreated cells (-). Relative expression ratios were measured respect to the sample transfected with the Negative Control miRNA Mimic and normalized by the expression levels of RPL13 for TRIM8 and p21, and by the expression level of U6 snRNA for miR-34a. * *p*-value < 0.05; ** *p*-value < 0.005; *** *p*-value < 0.001. (PDF 220 kb)
Additional file 9: Figure S8. (a, b) Expression levels of miR-17-5p and miR-106b-5p were measured by RT-qPCR in HCT116 cells, transfected with Negative Control miRNA Mimic, anti-miR-17-5p or anti-miR-106b-5p, and treated for 24 h with Nutlin-3 (N) (10 μM), Cisplatin (C) (7.5 μM), Sorafenib (S) (10 μM), Axitinib (A) (10 μM) or drug-untreated cells (-). Relative expression ratios were measured respect to the sample transfected with the Negative Control miRNA Mimic and normalized by the expression level of U6 snRNA. (c-e) Expression levels of TRIM8, p21, miR-34a and miR-17-5p were measured by RT-qPCR in the RCC-Shaw cells transfected with Negative Control miRNA Mimic or anti-miR-17-5p plus control short hairpin-RNA (control shRNA) or specific short hairpin against TRIM8 (shRNA-TRIM8). After transfection the cells were treated for 24 h with Nutlin-3 (N) (10 μM), Cisplatin (C) (7.5 μM), Sorafenib (S) (10 μM), Axitinib (A) (10 μM) or drug-untreated cells (-). Relative expression ratios were measured respect to the sample transfected with the Negative Control miRNA Mimic and normalized by the expression levels of RPL13 for TRIM8 and p21, and by the expression level of U6 snRNA for miR-34a and miR-17-5p. * *p*-value < 0.05; ** *p*-value < 0.005; *** *p*-value < 0.001 (f) Colony suppression assay. Cell growth were measured in HCT116 cells transfected with Negative Control miRNA Mimic, anti-miR-17-5p or anti-miR-106b-5p, and treated for 24 h with Nutlin-3 (N) (10 μM), Cisplatin (C) (7.5 μM), Sorafenib (S) (10 μM), Axitinib (A) (10 μM) or drug-untreated cells (-). (PDF 531 kb)
Additional file 10: Figure S9. (a-d) Expression levels of TRIM8, p21, miR-34a and miR-17-5p were measured by RT-qPCR in the RCC-Shaw cells transfected with Negative Control miRNA Mimic or anti-miR-17-5p plus control short hairpin-RNA (control shRNA) or specific short hairpin against TRIM8 (shRNA-TRIM8). After transfection the cells were treated for 24 h with Nutlin-3 (N) (10 μM), Cisplatin (C) (7.5 μM), Sorafenib (S) (10 μM), Axitinib (A) (10 μM) or drug-untreated cells (-). Relative expression ratios were measured respect to the sample transfected with the Negative Control miRNA Mimic and normalized by the expression levels of RPL13 for TRIM8 and p21, and by the expression level of U6 snRNA for miR-34a and miR-17-5p. ** *p*-value < 0.005; *** *p*-value < 0.001. (PDF 150 kb)
Additional file 11: Figure S10. (a) Representative xenograft tumours at the moment of the excision. (b) RT-qPCR demonstrating the expression of the exogenous TRIM8 or RING-TRIM8. The histogram shows the ratio between exogenous TRIM8 or RING-domain and the endogenous one. The analysis was conducted measuring the expression of the HA epitope and the expression of the total amount of RING domain in Control and Treated samples. The bars represent the Standard Error of the Mean. ***p*-value < 0.005. (PDF 650 kb)

## Abbreviations

BHD: Birt-Hogg-Dubé
Bta: Bos taurus
ccRCC: Clear cell renal cell carcinoma
CRC: Colorectal cancer
Dno: Dasypus novemcinctus
DTT: Dithiothreitol
Eeu: Erinaceus europaeus
Fca: Felis catus
Gga: Gallus gallus
GOI: Gene of interest
HKG: Housekeeping gene
Hsa: Homo sapiens
LOH: Loss of heterozigosity
Mdo: Monodelphis domestica
Mmu: Mus musculus
Oan: Ornithorhynchus anatinus
Ocu: Oryctolagus cuniculus
PBS: Phosphate-Buffered Saline
Ptr: Pan troglodytes
RIPA: Radioimmunoprecipitation assay
RT-qPCR: Reverse transcription quantitative polymerase chain reaction
shRNA: Short hairpin RNA
