# Supplementary figures and images for: TRIM8 restores p53 tumour suppressor function by blunting N-MYC activity in chemo-resistant tumours
Source: Mol Cancer. 2017 Mar 21;16:67. doi: 10.1186/s12943-017-0634-7 (PMC5359838; doi:10.1186/s12943-017-0634-7)

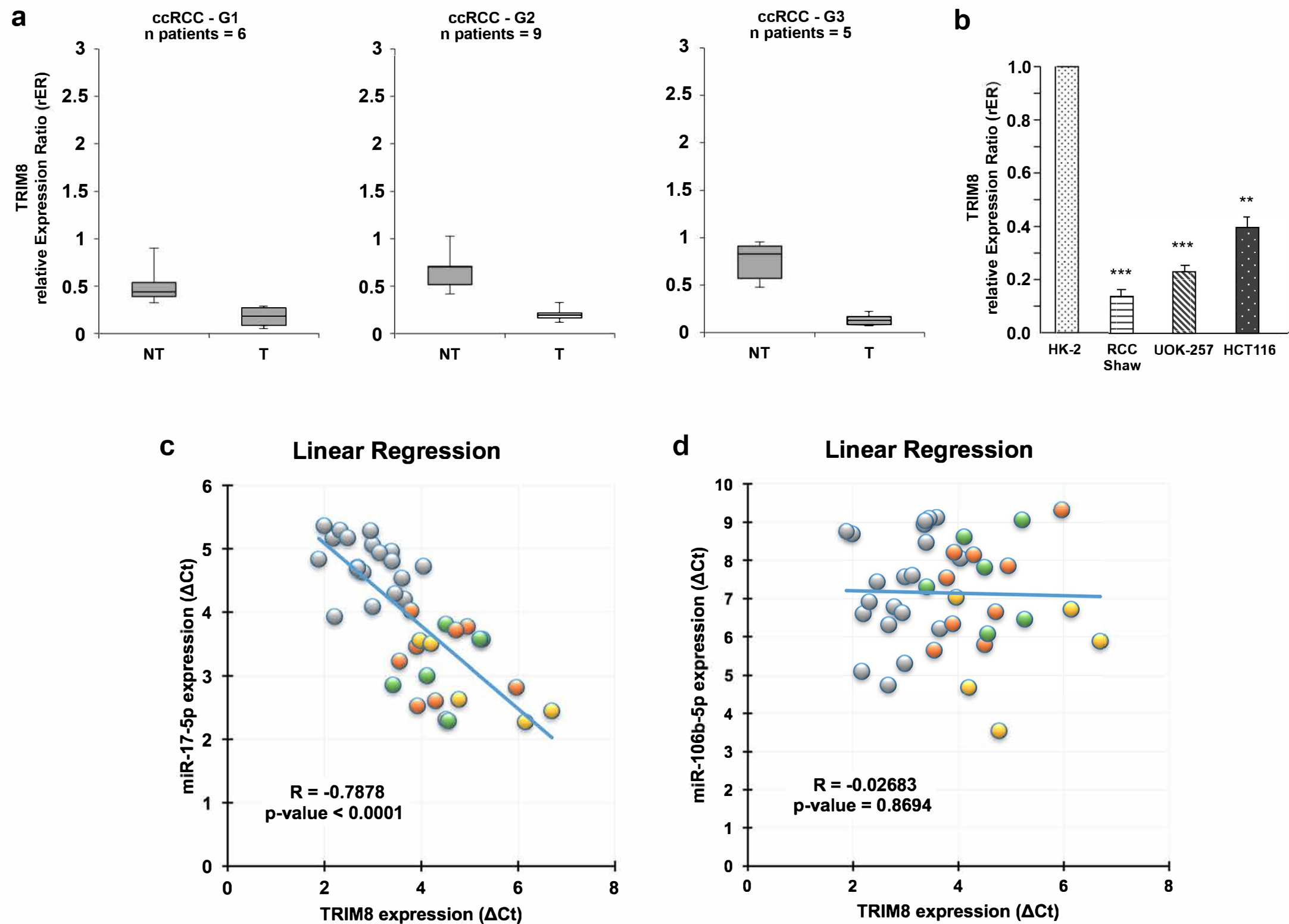

Figure S1

Supplement: Additional file 2: Figure S1. — (a) TRIM8 expression in ccRCC samples (T) and their paired non-tumour tissues (NT). The analysis was performed considering the Fuhrman grading of the tumour samples. Data are represented in box-and-whisker plots showing median and 10th, 25th, 75th and 90th percentiles for each category of sample. Expression data were measured respect to one normal sample chosen arbitrarily as calibrator and then normalized by the geometric mean of RPL13 and ACTB relative expression ratios. The bars represent the Standard Error of the Mean. (b) TRIM8 expression in three different renal cell lines: the human proximal tubular epithelial cells HK-2, the human renal cell carcinoma RCC-Shaw (p53wt) and the human renal carcinoma of BHD (Birt-Hogg-Dubè) origin UOK-257 cells (mutated-p53) and the colon cancer HCT116 (p53wt) cell line. Expression data were measured respect to HK-2 sample chosen as calibrator, and normalized by the expression levels of RPL13. **p-value < 0.005; *** p-value < 0.001. (c, d) Linear regression plots of miR-17-5p and miR-106b-5p expression levels compared with TRIM8 expression levels in ccRCC samples. On the x-axis the Ct of TRIM8 minus the arithmetic mean of Cts calculated for ACTB and RPL13 (used as housekeeping genes in RT-qPCR analysis) is reported, while the y-axis shows the Ct of miR-17-5p/miR-106b-5p minus the Ct of U6 snRNP (used to normalize miRNAs expression levels). The value of the correlation coefficient “r” and the p-value are indicated on the graph. Dots are coloured differentially according to the sample type: grey for non-tumour samples, green for Fuhrman Grade 1 samples, orange for Fuhrman Grade 2 samples and yellow for Fuhrman Grade 3 samples. (PDF 186 kb) [file 12943_2017_634_MOESM2_ESM.pdf]

**a**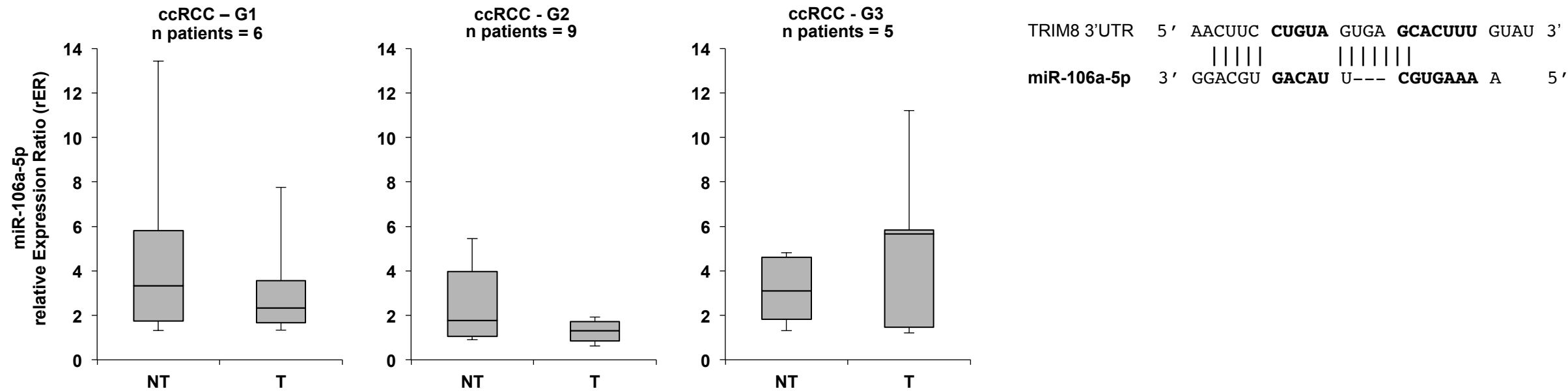**b**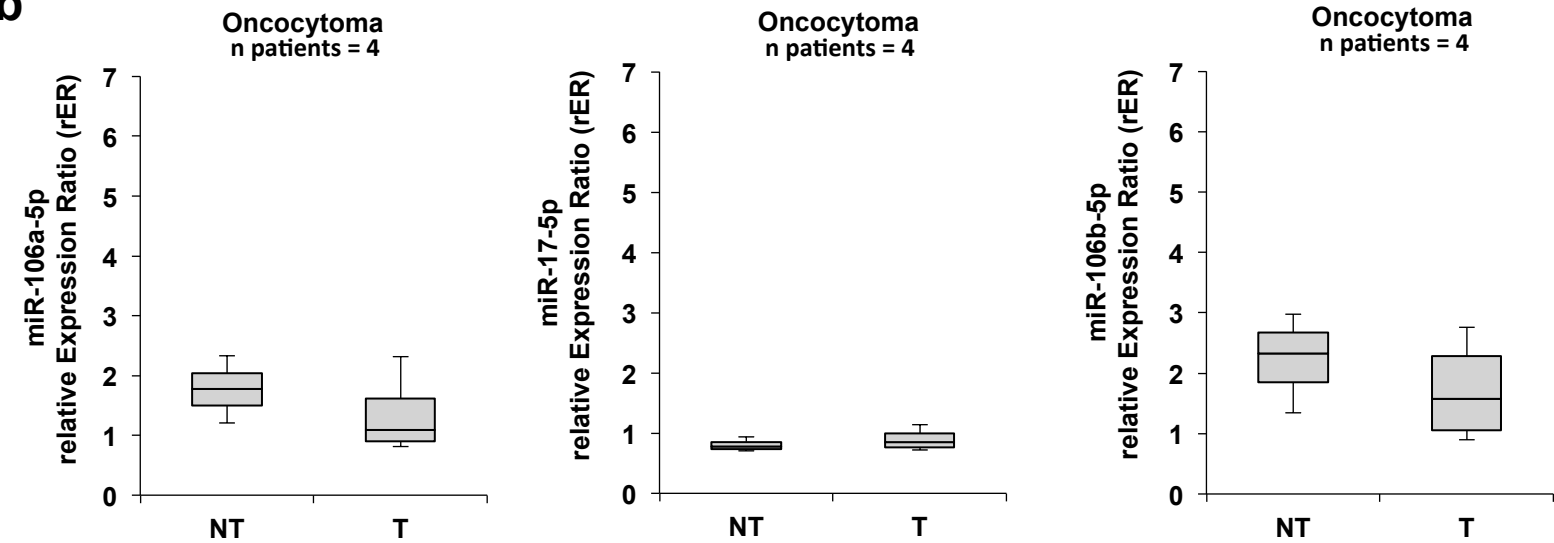

Figure S2

Supplement: Additional File 3: Figure S2. — (a) miR-106a-5p expression in ccRCC samples (T) and their paired non-tumour tissues (NT). The analysis was performed considering the Fuhrman grading of the tumour samples. Data are represented in box-and-whisker plots showing median and 10th, 25th, 75th and 90th percentiles for each category of sample. Expression data were measured respect to one normal sample chosen arbitrarily as calibrator and then normalized by the expression levels of U6 snRNA. The bars represent the Standard Error of the Mean. It is also reported the sequence alignment between the miR-106a-5p “seed” sequence and the TRIM8 3’UTR. (b) miR-106a-5p, miR-17-5p and miR-106b-5p expression in 4 oncocytoma samples (T) and their paired non-tumour tissues (NT). Data are represented in box-and-whisker plots showing median and 10th, 25th, 75th and 90th percentiles for each category of sample. Expression data were measured respect to one normal sample chosen arbitrarily as calibrator and then normalized by the expression levels of U6 snRNA. The bars represent the Standard Error of the Mean. (PDF 79 kb) [file 12943_2017_634_MOESM3_ESM.pdf]

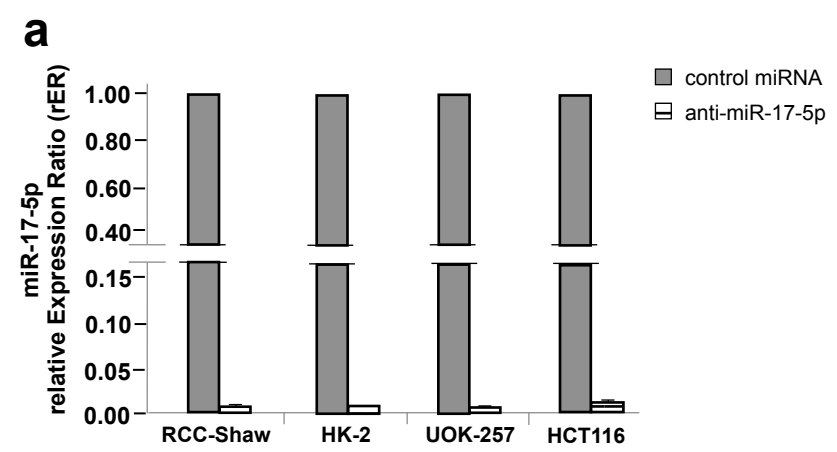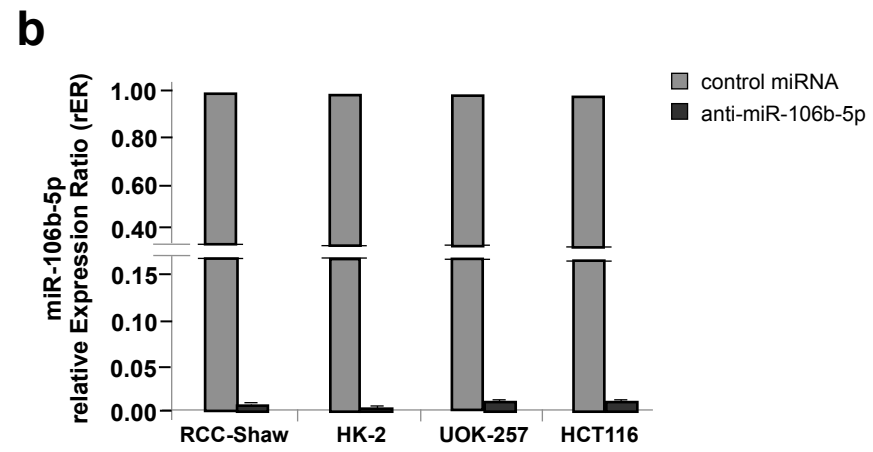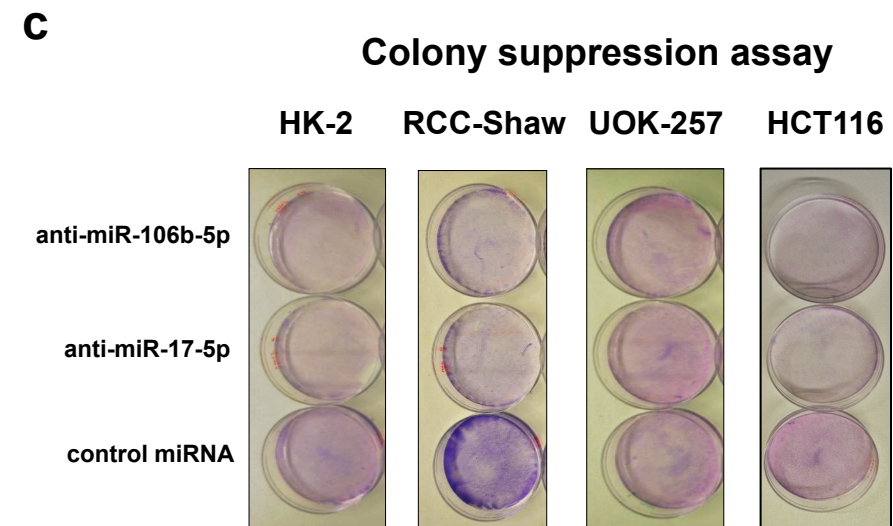

Figure S4

Supplement: Additional file 5: Figure S4. — (a, b) Expression levels of miR-17-5p and miR-106b-5p were measured by qPCR in HK-2, RCC-Shaw, UOK-257 and HCT116, transfected with Negative Control miRNA Mimic, anti-miR-17-5p or anti-miR-106b-5p. Relative expression ratios were measured respect to the sample transfected with the Negative Control miRNA Mimic and normalized by the expression level of U6 snRNA. The bars represent the Standard deviation of the Mean. (c) Colony suppression assays. Cell growth was measured in HK-2, RCC-Shaw, UOK-257 and HCT116 cells transfected with Negative Control miRNA Mimic, anti-miR-17-5p or anti-miR-106b-5p. (PDF 1350 kb) [file 12943_2017_634_MOESM5_ESM.pdf]

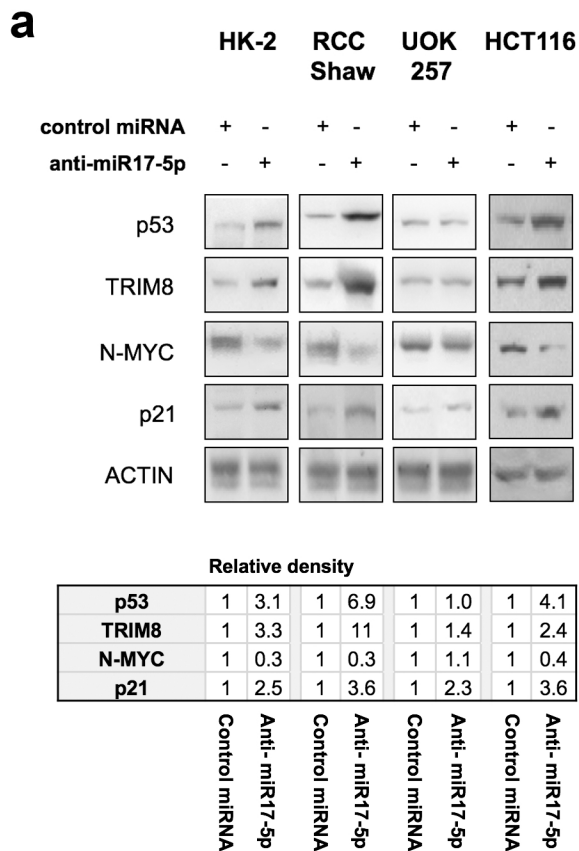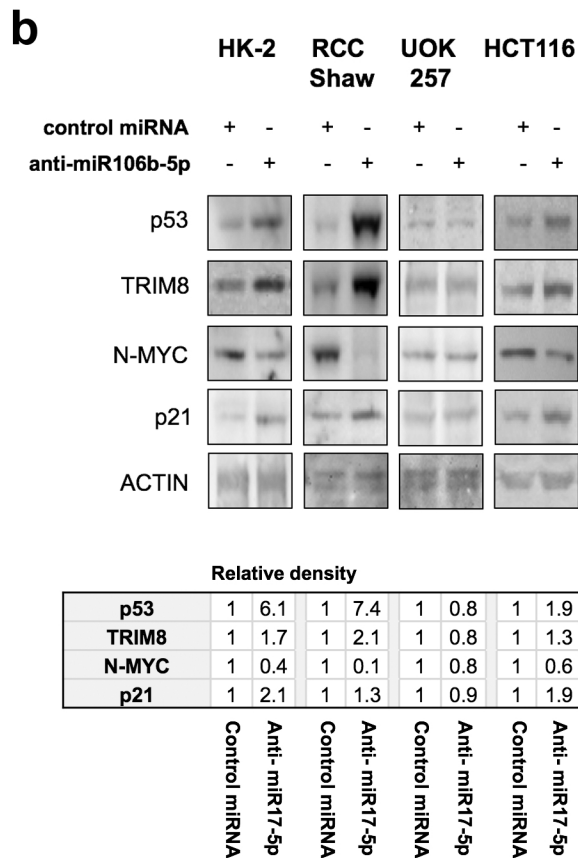

**Figure S5**

Supplement: Additional file 6: Figure S5. — (a-b) Densitometry analysis of p53, Trim8, N-MYC and p21 proteins in HK-2, UOK-257, RCC-Shaw and HCT116 cells transfected with negative control miRNA Mimic, anti-miR-17-5p (a) or anti-miR-106b-5p (b). The relative amounts of proteins, reported in the table below the western blotting, have been calculated normalizing to actin and calibrating to the respective control. (PDF 925 kb) [file 12943_2017_634_MOESM6_ESM.pdf]

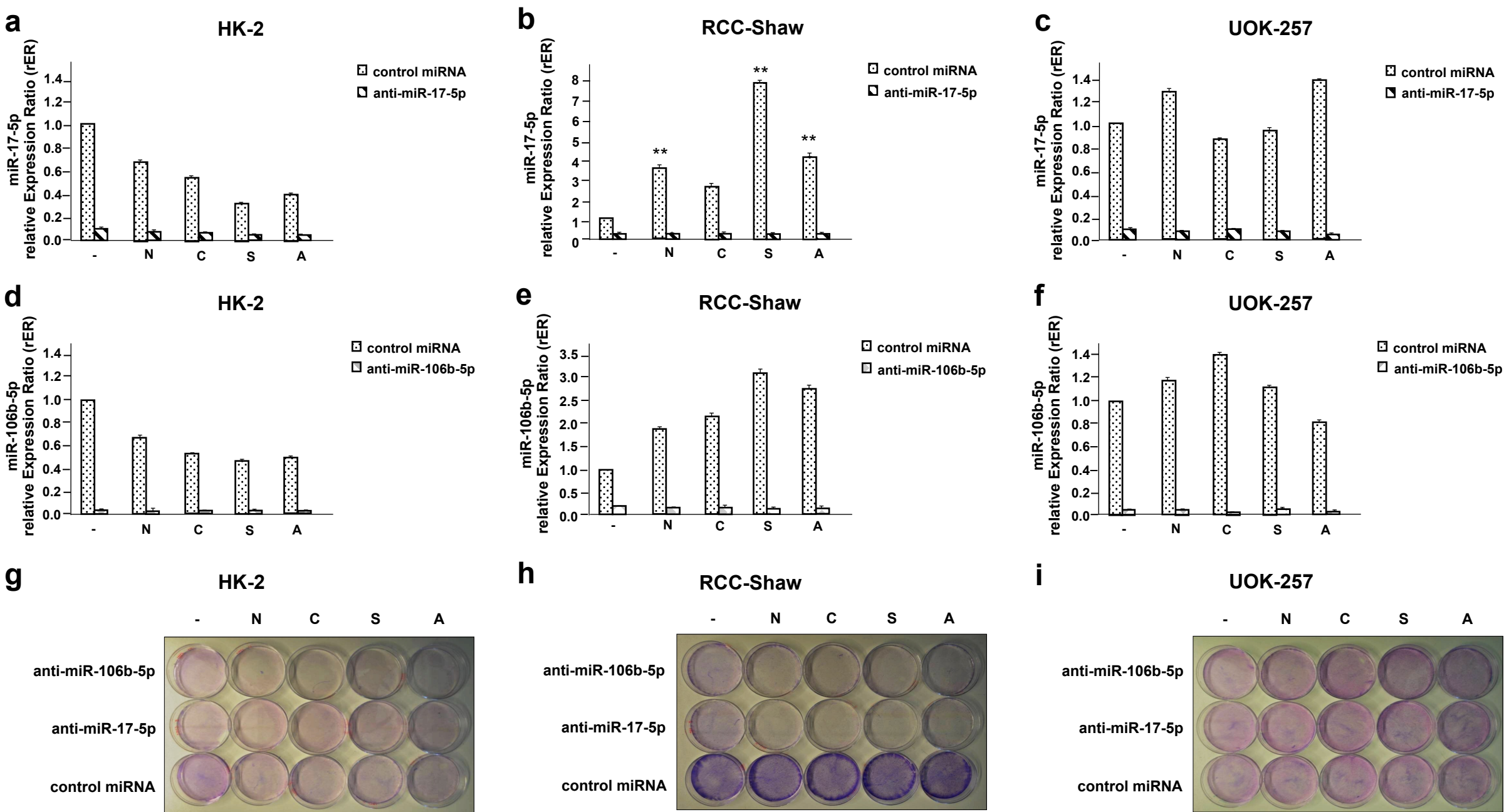

**Figure S6**

Supplement: Additional file 7: Figure S6. — (a-f) Expression levels of miR-17-5p and miR-106b-5p were measured by RT-qPCR in HK-2, RCC-Shaw and UOK-257 cells, transfected with Negative Control miRNA Mimic, anti-miR-17-5p or anti-miR-106b-5p, and treated for 24 h with Nutlin-3 (N) (10 μM), Cisplatin (C) (7.5 μM), Sorafenib (S) (10 μM), Axitinib (A) (10 μM) or drug-untreated cells (-). Relative expression ratios were measured respect to the sample transfected with the Negative Control miRNA Mimic and normalized by the expression level of U6 snRNA. ** p-value < 0.005 (g-i) Colony suppression assay. Cell growth were measured in HK-2, RCC-Shaw and UOK-257 cells transfected with Negative Control miRNA Mimic, anti-miR-17-5p or anti-miR-106b-5p, and treated for 24 h with Nutlin-3 (N) (10 μM), Cisplatin (C) (7.5 μM), Sorafenib (S) (10 μM), Axitinib (A) (10 μM) or drug-untreated cells (-). (PDF 380 kb) [file 12943_2017_634_MOESM7_ESM.pdf]

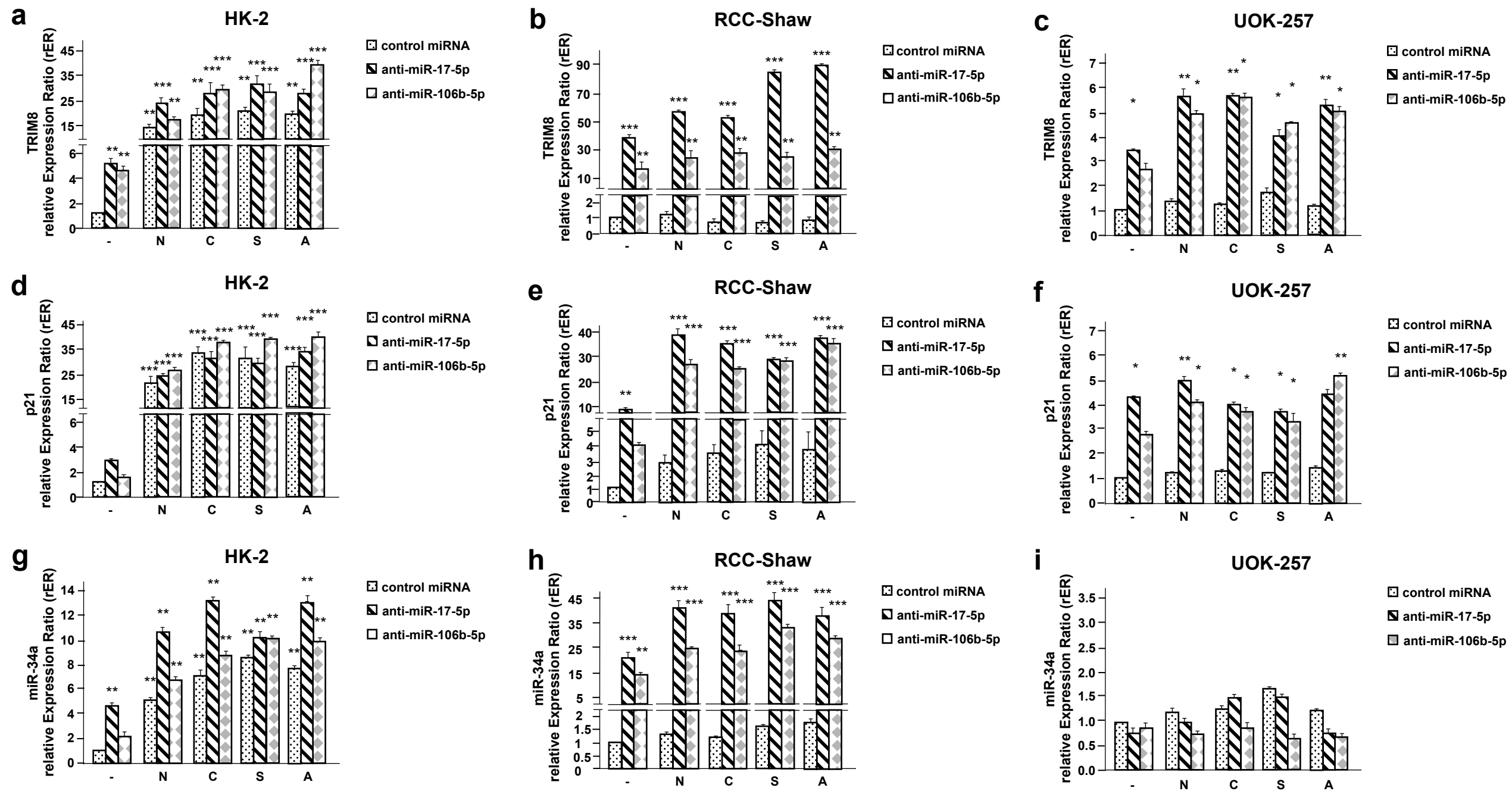

**Figure S7**

Supplement: Additional file 8: Figure S7. — (a-i) Expression levels of TRIM8, p21 and miR-34a were measured by RT-qPCR in HK-2, RCC-Shaw and UOK-257 cells, transfected with Negative Control miRNA Mimic, anti-miR-17-5p or anti-miR-106b-5p, and treated for 24 h with Nutlin-3 (N) (10 μM), Cisplatin (C) (7.5 μM), Sorafenib (S) (10 μM), Axitinib (A) (10 μM) or drug-untreated cells (-). Relative expression ratios were measured respect to the sample transfected with the Negative Control miRNA Mimic and normalized by the expression levels of RPL13 for TRIM8 and p21, and by the expression level of U6 snRNA for miR-34a. * p-value < 0.05; ** p-value < 0.005; *** p-value < 0.001. (PDF 220 kb) [file 12943_2017_634_MOESM8_ESM.pdf]

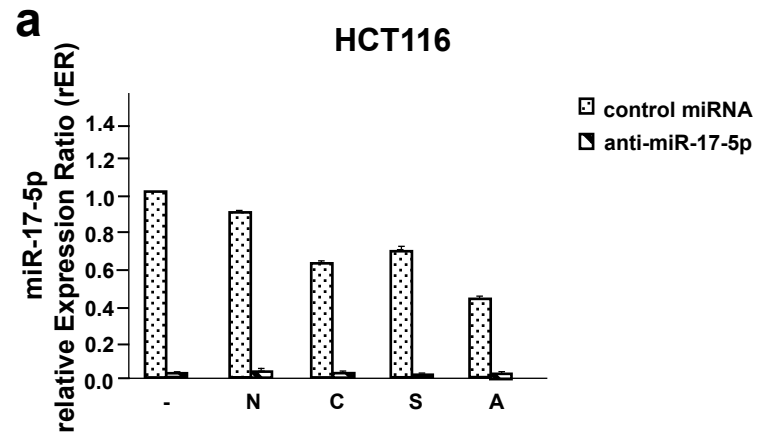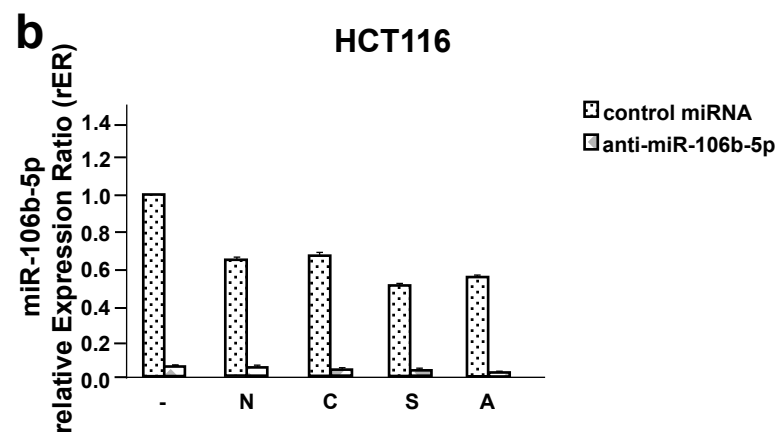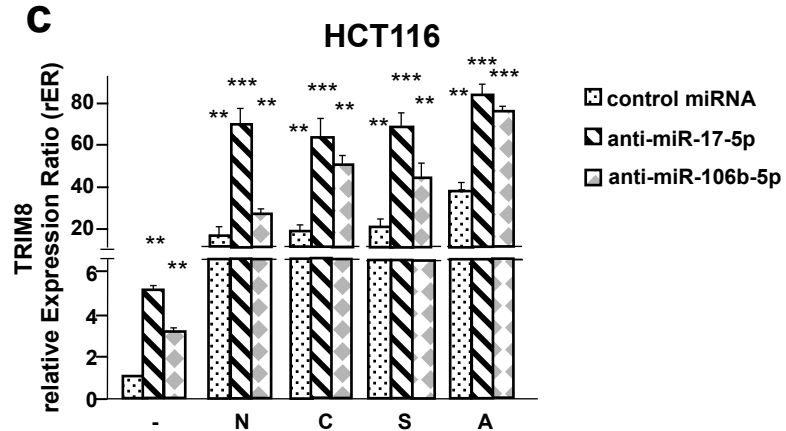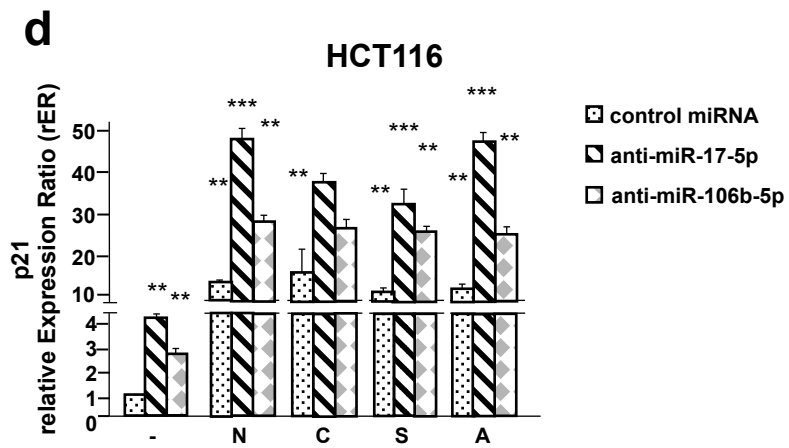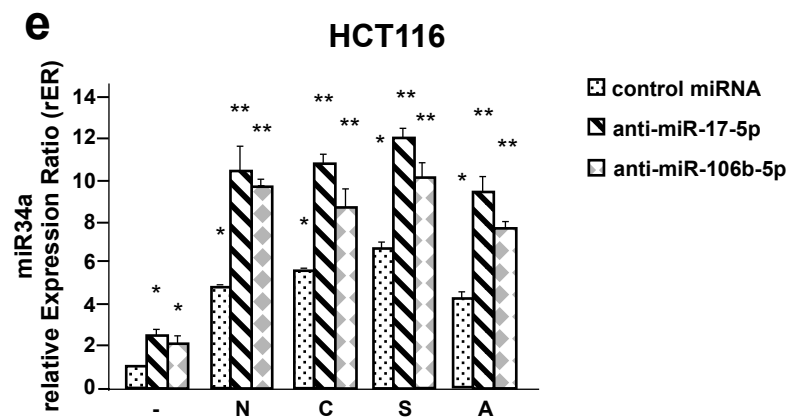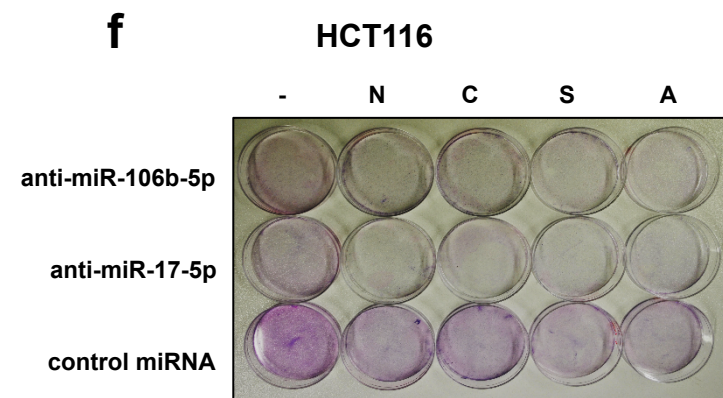

Figure S8

Supplement: Additional file 9: Figure S8. — (a, b) Expression levels of miR-17-5p and miR-106b-5p were measured by RT-qPCR in HCT116 cells, transfected with Negative Control miRNA Mimic, anti-miR-17-5p or anti-miR-106b-5p, and treated for 24 h with Nutlin-3 (N) (10 μM), Cisplatin (C) (7.5 μM), Sorafenib (S) (10 μM), Axitinib (A) (10 μM) or drug-untreated cells (-). Relative expression ratios were measured respect to the sample transfected with the Negative Control miRNA Mimic and normalized by the expression level of U6 snRNA. (c-e) Expression levels of TRIM8, p21, miR-34a and miR-17-5p were measured by RT-qPCR in the RCC-Shaw cells transfected with Negative Control miRNA Mimic or anti-miR-17-5p plus control short hairpin-RNA (control shRNA) or specific short hairpin against TRIM8 (shRNA-TRIM8). After transfection the cells were treated for 24 h with Nutlin-3 (N) (10 μM), Cisplatin (C) (7.5 μM), Sorafenib (S) (10 μM), Axitinib (A) (10 μM) or drug-untreated cells (-). Relative expression ratios were measured respect to the sample transfected with the Negative Control miRNA Mimic and normalized by the expression levels of RPL13 for TRIM8 and p21, and by the expression level of U6 snRNA for miR-34a and miR-17-5p. * p-value < 0.05; ** p-value < 0.005; *** p-value < 0.001 (f) Colony suppression assay. Cell growth were measured in HCT116 cells transfected with Negative Control miRNA Mimic, anti-miR-17-5p or anti-miR-106b-5p, and treated for 24 h with Nutlin-3 (N) (10 μM), Cisplatin (C) (7.5 μM), Sorafenib (S) (10 μM), Axitinib (A) (10 μM) or drug-untreated cells (-). (PDF 531 kb) [file 12943_2017_634_MOESM9_ESM.pdf]

# RCC-Shaw

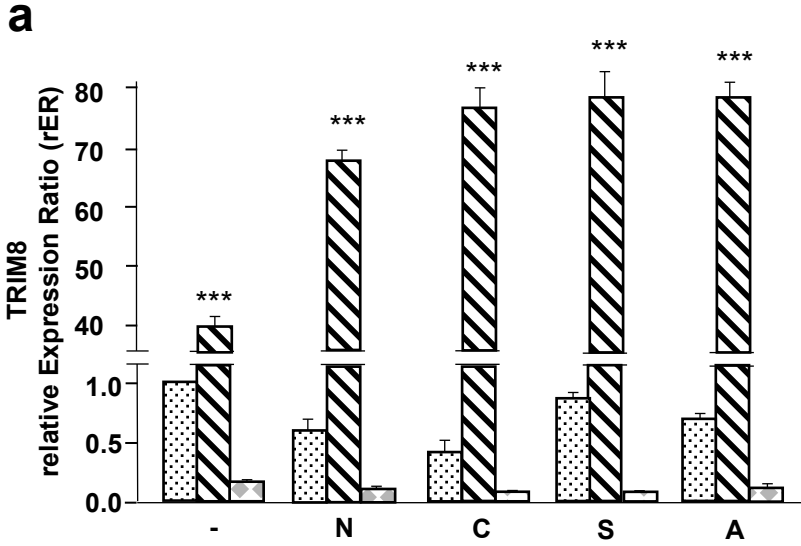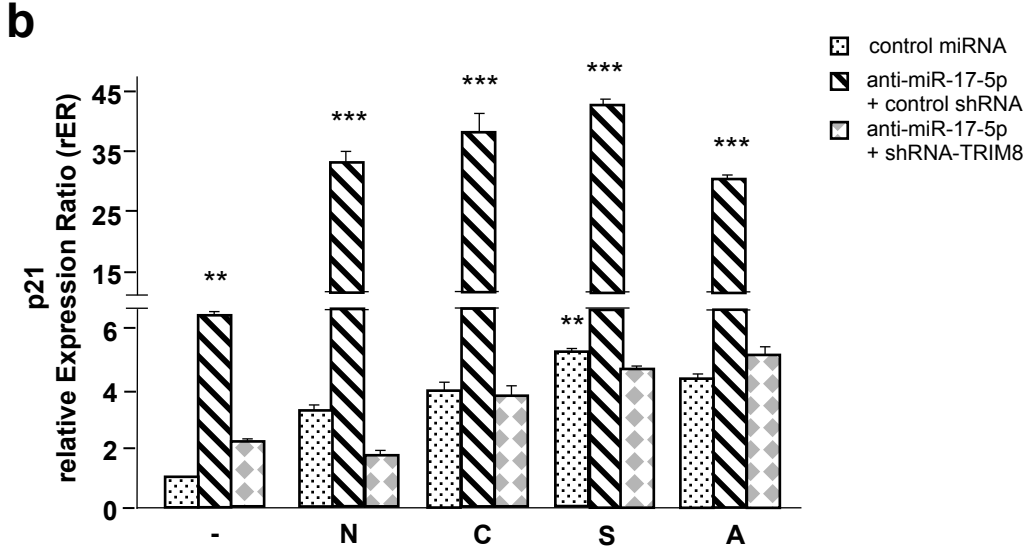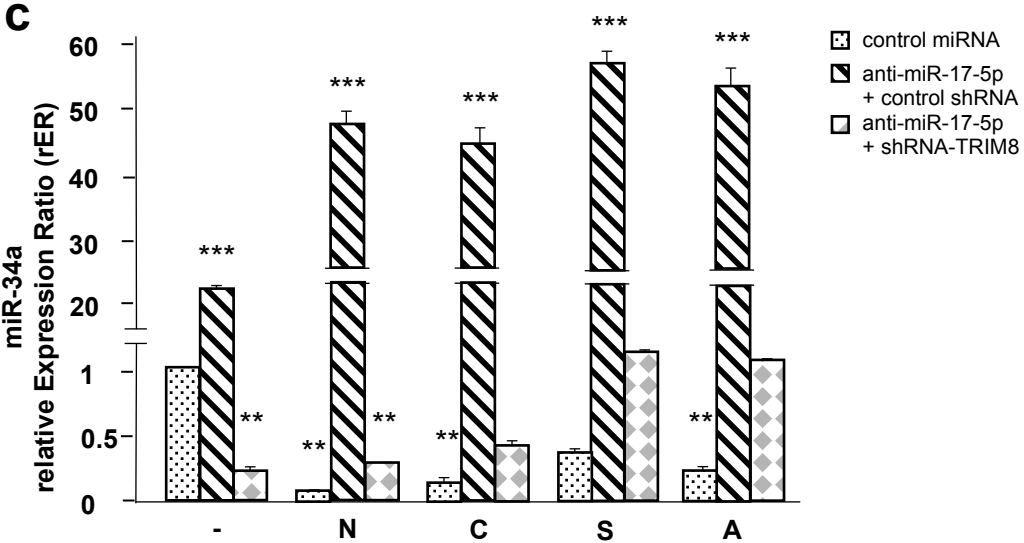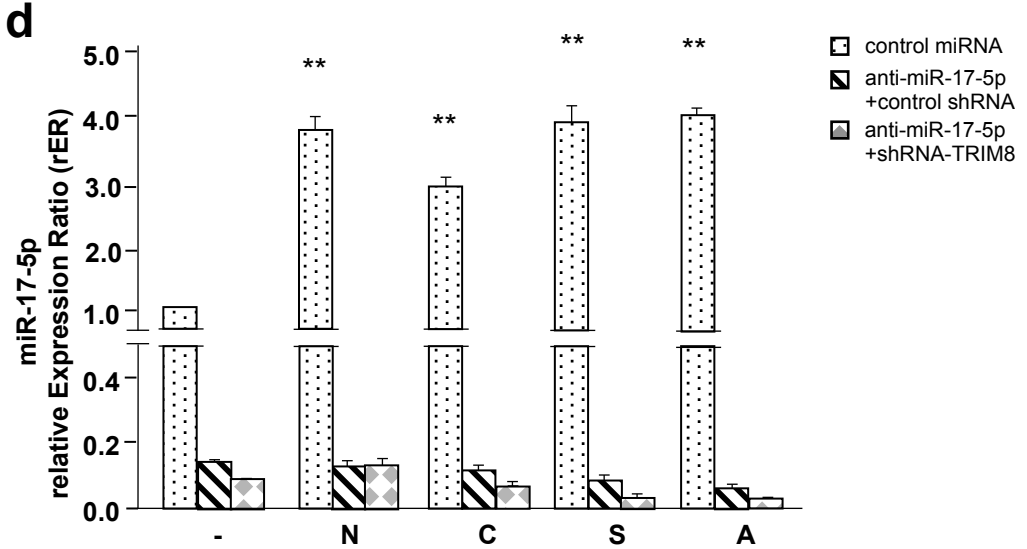

## Figure S9

Supplement: Additional file 10: Figure S9. — (a-d) Expression levels of TRIM8, p21, miR-34a and miR-17-5p were measured by RT-qPCR in the RCC-Shaw cells transfected with Negative Control miRNA Mimic or anti-miR-17-5p plus control short hairpin-RNA (control shRNA) or specific short hairpin against TRIM8 (shRNA-TRIM8). After transfection the cells were treated for 24 h with Nutlin-3 (N) (10 μM), Cisplatin (C) (7.5 μM), Sorafenib (S) (10 μM), Axitinib (A) (10 μM) or drug-untreated cells (-). Relative expression ratios were measured respect to the sample transfected with the Negative Control miRNA Mimic and normalized by the expression levels of RPL13 for TRIM8 and p21, and by the expression level of U6 snRNA for miR-34a and miR-17-5p. ** p-value < 0.005; *** p-value < 0.001. (PDF 150 kb) [file 12943_2017_634_MOESM10_ESM.pdf]

**a**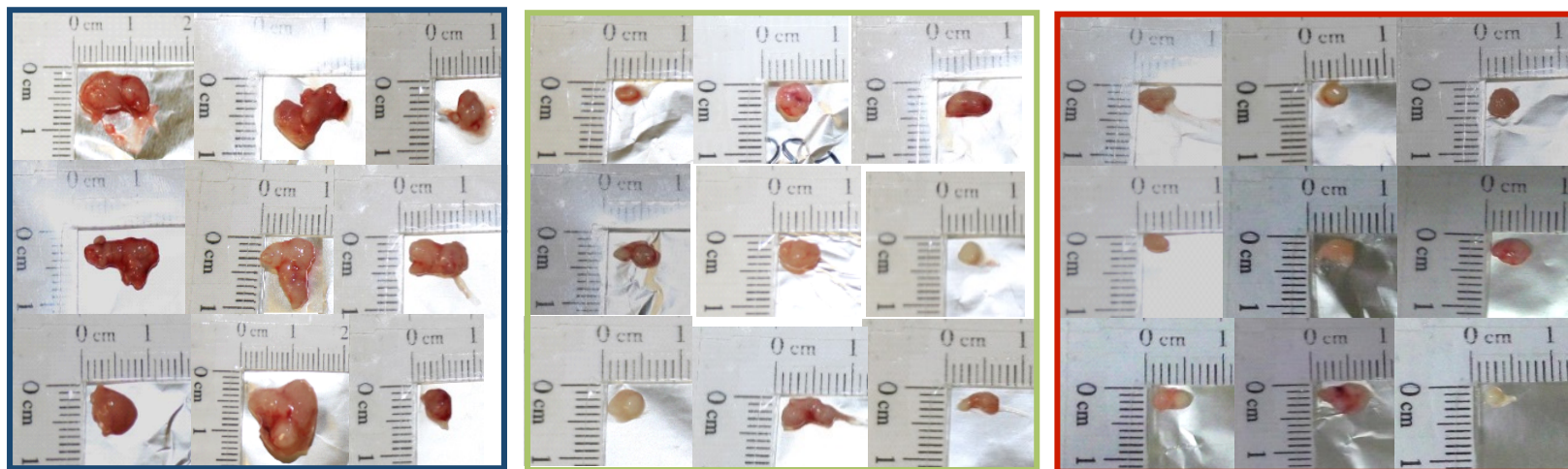

Control

Treated with HA-TRIM8

Treated with HA-RING-TRIM8

**b**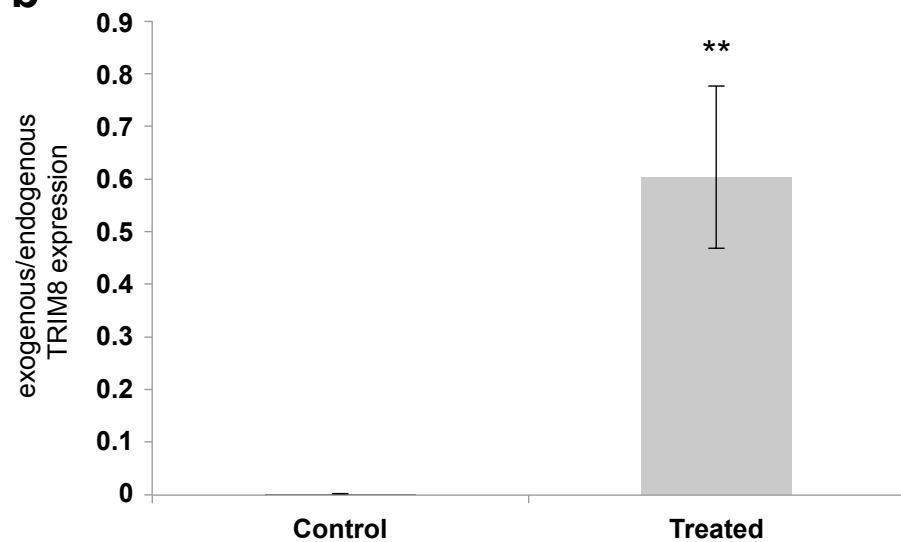**Figure S10**

Supplement: Additional file 11: Figure S10. — (a) Representative xenograft tumours at the moment of the excision. (b) RT-qPCR demonstrating the expression of the exogenous TRIM8 or RING-TRIM8. The histogram shows the ratio between exogenous TRIM8 or RING-domain and the endogenous one. The analysis was conducted measuring the expression of the HA epitope and the expression of the total amount of RING domain in Control and Treated samples. The bars represent the Standard Error of the Mean. **p-value < 0.005. (PDF 650 kb) [file 12943_2017_634_MOESM11_ESM.pdf]
