# Supplementary material for: TRIM8 restores p53 tumour suppressor function by blunting N-MYC activity in chemo-resistant tumours
Source: Mol Cancer. 2017 Mar 21;16:67. doi: 10.1186/s12943-017-0634-7 (PMC5359838; doi:10.1186/s12943-017-0634-7)
Supplement: Additional file 4: Figure S3. — (a, b) Luciferase assay. The HK-2 and the HCT116 cells were transfected with Negative Control miRNA Mimic, miR-17-5p, miR-106b-5p, anti-miR-17-5p or anti-miR-106b-5p, along with pMIR luciferase reporter construct containing p21 3’UTR. Cells were lysed and luciferase activity was determined as described in the Material and Methods section. Transfection efficacy was normalized by Renilla Luciferase activity. Data represent the averages of at least three independent experiments with their standard deviations. ** p-value < 0.005; *** p-value < 0.001. (PDF 64 kb) [file 12943_2017_634_MOESM4_ESM.pdf]

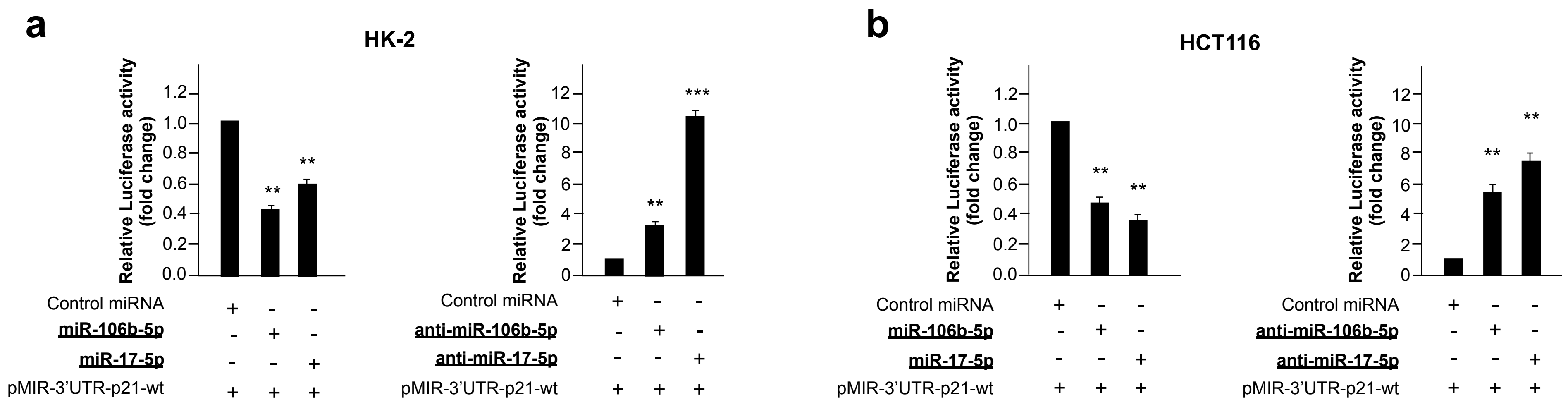
a
HK-2
Relative Luciferase activity (fold change)
1.2
1.0
0.8
0.6
0.4
0.2
0.0
**
**
Control miRNA + - -
miR-106b-5p - + -
miR-17-5p - - +
pMIR-3'UTR-p21-wt + + +
Relative Luciferase activity (fold change)
12
10
8
6
4
2
0
**
***
Control miRNA + - -
anti-miR-106b-5p - + -
anti-miR-17-5p - - +
pMIR-3'UTR-p21-wt + + +
b
HCT116
Relative Luciferase activity (fold change)
1.2
1.0
0.8
0.6
0.4
0.2
0.0
**
**
Control miRNA + - -
miR-106b-5p - + -
miR-17-5p - - +
pMIR-3'UTR-p21-wt + + +
Relative Luciferase activity (fold change)
12
10
8
6
4
2
0
**
**
Control miRNA + - -
anti-miR-106b-5p - + -
anti-miR-17-5p - - +
pMIR-3'UTR-p21-wt + + +

Figure S3
